# Low-dimensional solid-state single-photon emitters

**DOI:** 10.1515/nanoph-2024-0569

**Published:** 2025-01-08

**Authors:** Jinli Chen, Chaohan Cui, Ben Lawrie, Yongzhou Xue, Saikat Guha, Matt Eichenfield, Huan Zhao, Xiaodong Yan

**Affiliations:** Department of Materials Science and Engineering, 8041 University of Arizona, Tucson, AZ 85721, USA; Department of Electrical and Computer Engineering, University of Maryland, College Park, MD 20742, USA; Center for Nanophase Materials Sciences, Oak Ridge National Laboratory, Oak Ridge, TN 37831, USA; Materials Sciences and Technology Division, Oak Ridge National Laboratory, Oak Ridge, TN 37831, USA; James C. Wyant College of Optical Sciences, 8041 University of Arizona, Tucson, AZ 85721, USA; Department of Electrical and Computer Engineering, 8041 University of Arizona, Tucson, AZ 85721, USA

**Keywords:** low-dimensional materials, single photon sources, quantum dots, single-walled carbon nanotubes, transition metal dichalcogenides, hexagonal boron nitride

## Abstract

Solid-state single-photon emitters (SPEs) are attracting significant attention as fundamental components in quantum computing, communication, and sensing. Low-dimensional materials-based SPEs (LD-SPEs) have drawn particular interest due to their high photon extraction efficiency, ease of integration with photonic circuits, and strong coupling with external fields. The accessible surfaces of LD materials allow for deterministic control over quantum light emission, while enhanced quantum confinement and light–matter interactions improve photon emissive properties. This perspective examines recent progress in LD-SPEs across four key materials: zero-dimensional (0D) semiconductor quantum dots, one-dimensional (1D) nanotubes, two-dimensional (2D) materials, including hexagonal boron nitride (hBN) and transition metal dichalcogenides (TMDCs). We explore their structural and photophysical properties, along with techniques such as spectral tuning and cavity coupling, which enhance SPE performance. Finally, we address future challenges and suggest strategies for optimizing LD-SPEs for practical quantum applications.

## 1 Introduction

Photonic quantum technologies that harness the quantum properties of light (photons) to process quantum information have drawn increasing interest over the past decade. Single-photon sources [1], [2], [3], [4] – isolated quantum systems designed to emit precisely one photon per excitation cycle – have garnered widespread research interest. These flying qubits play a crucial role in encoding, transmitting, and transducing quantum information, forming the foundation of many approaches to quantum computing [5], metrology [6], sensing [7], and secure communication [8].

Solid-state SPEs stand out by combining the application-specific tailoring of optical properties, such as wavelength, intensity, and polarization, while maintaining the scalability of solid-state systems [9], [10], [11], offering on-demand emission that is not available in other quantum light sources, such as those utilizing nonlinear optical techniques like spontaneous parametric down-conversion (SPDC) [12], [13], [14] and spontaneous four-wave mixing (SFWM) [15]. SPEs have been reported in various material platforms such as quantum dots (QDs) [16], [17], rare earth ions [18], [19], [20], and defect centers [21] (e.g., nitrogen-vacancy (NV) centers in diamond [22], [23], [24]), offering high brightness, purity, and indistinguishability. Additionally, some SPEs exhibit optically addressable spin states, suitable for quantum sensing, transduction, and nuclear magnetic resonance spectroscopy [25].

SPEs in 3D bulk materials such as silicon [26], [27] and diamond suffer from low (<10 %) intrinsic photon extraction efficiency due to internal reflection, although extensive efforts have gone into patterning 3D photonic platforms with improved extraction efficiency [28]. In addition, integrating 3D SPEs into photonic structures [29] is usually not straightforward and requires demanding fabrication process [30], [31], [32]. LD-materials, on the other hand, offer high photon extraction efficiencies and comparably easy integration with photonic interfaces [33], [34], [35]. Their accessible surface and enhanced surface-volume ratio allows for deterministic SPE creation [36], [37] and efficient tuning of emission properties [38], [39]. Additionally, low-dimensional materials could host exotic physical phenomena, such as valley degrees of freedom [40] and exciton–magnon coupling [41], [42], which pave the way for coherent control of photonic qubits via external fields [43], [44].

**Figure 1:**
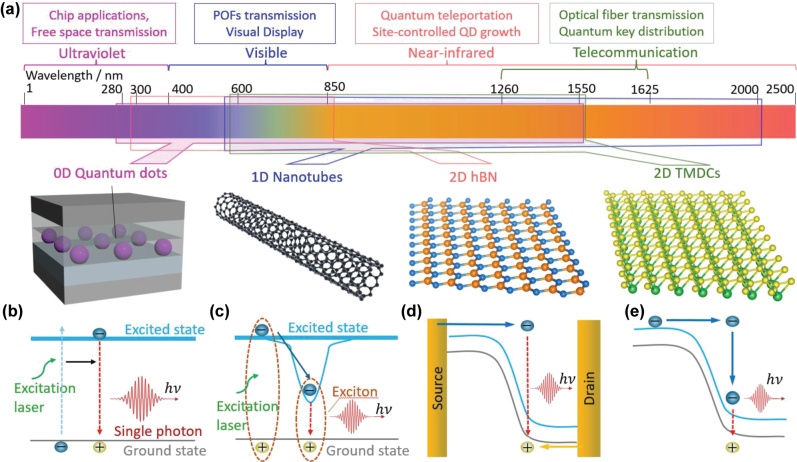
Wavelength centric overview of low-dimensional materials based SPEs. (a) Electromagnetic spectrum showing spectral ranges and applications for SPEs across ultraviolet, visible, near-infrared, and telecommunication wavelengths, from left to right. The top portion highlights applications, while the bottom shows schematic illustrations of SPE materials: quantum dots, nanotubes, hBN, and TMDCs. Colored polygons indicate the spectral ranges covered by each material. (b)–(e) Different mechanisms for generating single photons. (b) Spontaneous decay of excited states, where an excitation laser promotes an electron to the excited state, and single photons are emitted during relaxation. (c) Spontaneous decay of localized excitons, where the laser creates excitons, which recombine to emit single photons. (d) Ambipolar emission in electroluminescent devices, where electron–hole recombination between the source and drain generates photons. (e) Unipolar emission mechanism via impact excitation in electroluminescent devices, where high-energy carriers excite electrons to emit photons during relaxation.

This review examines the advancement in LD-SPEs developed from four key material systems: 0D semiconductor QDs, 1D nanotubes, 2D hBN, and 2D TMDCs (Figure 1a). We begin by discussing the mechanism behind single-photon emission (Section 2) and the characterization of SPEs (Section 3). This is followed by an in-depth review of the structures, photophysical properties, state-of-the-art performance, applications, and challenges associated with LD-SPEs (Sections 4–7). In Sections 8 and 9, we explore techniques like spectral tuning and cavity coupling, which are widely utilized to enhance SPE performance. Finally, in Section 10, we summarize the key findings in LD-SPEs and provide perspectives on future research directions.

## 2 Single-photon emitters: realization and excitation

Over the past four decades, SPEs have been successfully demonstrated in a wide variety of materials. These include bulk materials such as diamond [45], [46], silicon carbide (SiC) [47], silicon nitride (SiN) [48], aluminum nitride (AlN) [49], [50], and gallium nitride (GaN) [51], [52]; two-dimensional materials like hBN [53], [54] and tungsten diselenide (WSe_2_) [55], [56], [57], [58]; one-dimensional materials like single-walled carbon nanotubes (SWCNTs) [59], [60] and nanowires [61]; and zero-dimensional materials such as colloidal quantum dots [62], graphene quantum dots [33], and epitaxial quantum dots [63], [64]. Single-photon emission in these materials typically originates from two mechanisms: (1) color centers and (2) excitons confined within nanostructures or potential wells [34]. In both cases, a well-defined two-level energy structure is required to avoid unwanted emission from multiple optical transitions [65].

Color centers, usually observed in wide-bandgap systems such as diamond [31] and hBN [66], are defects in the crystal lattice where an atom is either missing or replaced by a different atom (i.e., vacancies or impurities). They disrupt the periodic potential within the solid, creating localized electronic states within the material’s bandgap (Figure 1b). These defects, which are usually found in nanocrystals, exfoliated layers, powders, and bulk materials, can be created through thermal annealing [67], femtosecond laser direct writing [68], electron or ion beam irradiation [69], [70], mechanical indentation [71], and chemical/plasma treatment [72], [73]. However, these techniques face physical limitations that hinder the consistent creation of identical emitters and frequently lead to the formation of unintended defects, thereby impeding the production of uniform SPEs.

Confined excitons, usually observed in LD materials such QDs [17], SWCNTs [74], and TMDCs [75], are bound states of excitons that are spatially localized (Figure 1c). These regions, often caused by defects, strain, or size confinement, disrupt the uniform electronic potential, resulting in quantized energy states [76] that can be engineered through mechanical exfoliation [56], chemical vapor deposition (CVD) [77], chemical functionalization [78], and strain engineering [79]. However, these methods may result in spatial and spectral inhomogeneities due to intrinsic solid-state environment noise and fluctuations in fabrication conditions, complicating the generation of reproducible and indistinguishable SPEs. Thus, new techniques that can inherently ensure the production of identical SPEs are highly desired.

Excitation involves pumping electrons from the ground state to an excited state. We categorize the methods for exciting LD-SPEs based on various perspectives, including optical versus electrical excitation, continuous wave (CW) versus pulsed excitation, and resonant versus nonresonant excitation, as detailed below.

### 2.1 Optical versus electrical excitation

**Figure 2:**
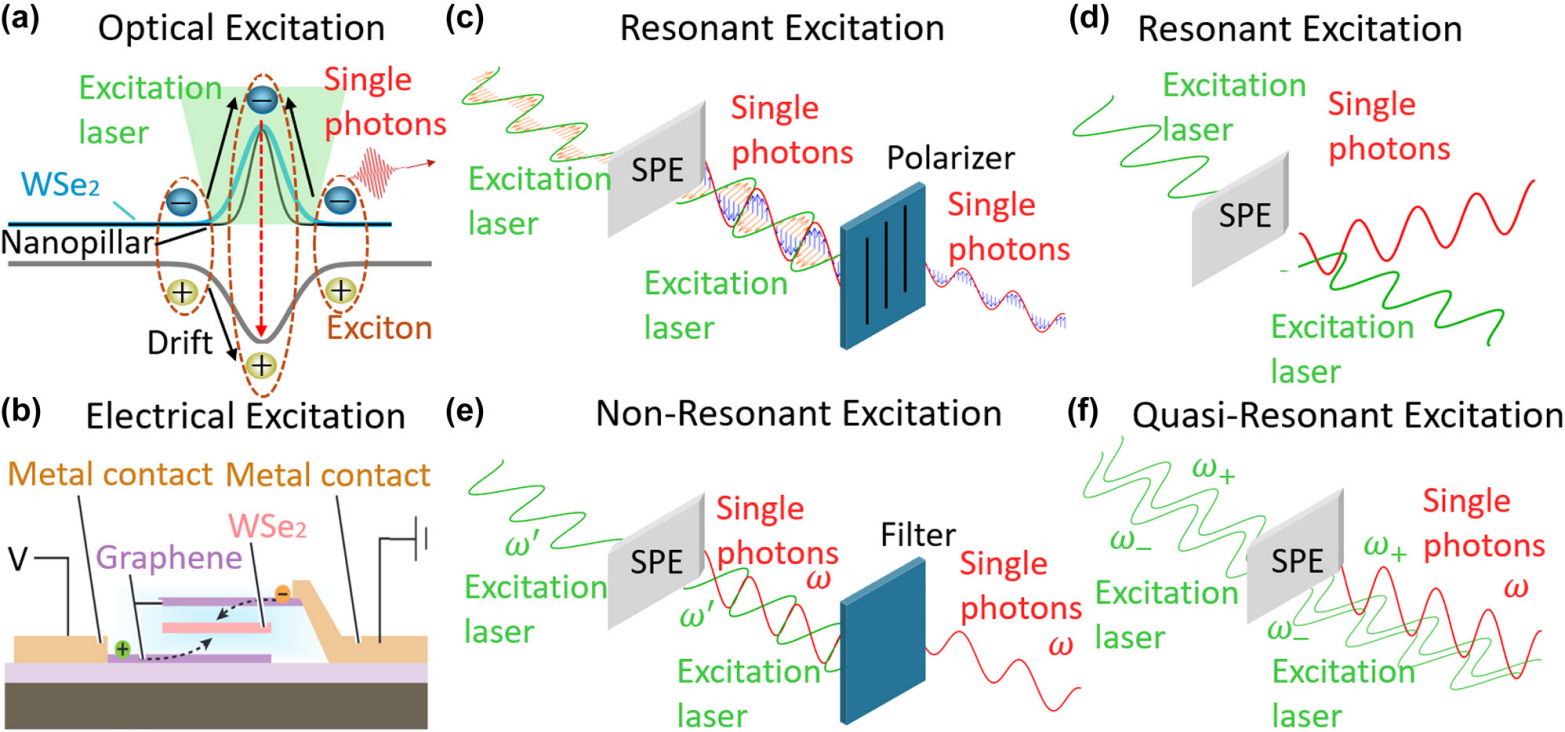
Different excitation schemes in 2D SPEs. (a) Schematic of the optical excitation mechanism in WSe_2_-based SPEs. An excitation laser excites electrons from the ground state to the excited state, forming excitons in the WSe_2_ layer positioned on a nanopillar. The excitons drift, and the electron–hole recombination results in the emission of single photons via spontaneous emission. (b) A vertical electrical excitation device comprised of WSe_2_ sandwiched between few-layer boron nitride and metal contacts. The device structure includes graphene as a conductive layer, with electrical excitation applied through metal contacts to generate excitons within the WSe_2_ layer. (c) Resonant excitation. The excitation laser and emitted single photons are spectrally filtered based on their polarization. (d) Resonant excitation, spectrally filtering is applied to separate the propagation direction of the excitation laser and the emitted photons. (e) Nonresonant excitation. The excitation laser (*ω*′) and emitted single photons (*ω*) have different wavelengths. Spectral filtering is applied to isolate the single photons from the excitation laser based on their distinct wavelengths. (f) A kind of quasi-resonant excitation. Two near-resonant excitation laser pulses (*ω*_+_ and *ω*_−_) are used to predictably manipulate the emitter into its excited state, leading to the emission of single photons (*ω*). Adapted with permission from: (b), ref. [81] Copyright 2016, American Chemical Society.

Optical excitation is widely used for SPEs and can be controlled by adjusting light intensity and wavelength. Jungwirth et al. [80] employed optical excitation to survey the temperature-dependent emission of point defects in multilayer hBN. They identified single-photon emission from individual SPEs by leveraging the spectral selectivity and high spatial resolution of optical excitation. Electrical excitation, alternatively, uses an electric current or voltage to stimulate SPEs, enabling single-photon emission through ambipolar emission (Figure 1d), where both electrons and holes recombine, or unipolar emission (Figure 1e), where a single type of carrier recombines with existing states. Clark et al. [81] demonstrated electrically driven SPEs in WSe_2_ and observed their electroluminescence (EL) intensities at cryogenic temperature, paving the way for on-chip SPEs in TMDCs. Figure 2a and b illustrate the different mechanisms of optical and electrical excitations.

Cathodoluminescence (CL) microscopies have separately emerged as a powerful resource for near-field imaging of LD-SPEs [82], [83], [84], [85], [86], [87], [88], [89]. CL microscopy leverages converged electron-beams in scanning (transmission) electron microscopes combined with far-field optical detection of photons generated by the material after electron-beam excitation in order to probe LD-SPEs with true nanoscale resolution well below the optical diffraction limit. While CL microscopies have been used to probe the photon statistics of SPEs, much of the literature exhibits photon bunching instead of photon antibunching under electron-beam excitation because the high-energy electron-beam can easily excite unwanted electronic transitions, resulting in the concurrent emission of photons from many excited states at once [83], [84], [90], [91]. However, appropriate choices of electron-beam current do allow for nanoscale probes of photon antibunching [87], [88], [92]. CL microscopy is a particularly appealing tool for LD-SPEs because of the potential for *in situ* patterning and modification of SPEs and their environments. For instance, CL microscopy has been used for *in situ* monitoring of e-beam patterned defects in hBN [86], [89], and e-beam induced etching in water vapor environments has been used to pattern nanoscale diamond cavities with *in situ* CL feedback [32]. Ultimately, the ability to image and pattern LD-SPEs at these length scales may lead to the development of new integrated quantum photonic systems with optimized cavity interactions designed to achieve ideal SPE properties.

### 2.2 Continuous wave (CW) versus pulsed excitation

CW excitation, which uses a steady energy source such as a laser or electric current, enables sustained single-photon emission but can reduce purity at higher excitation power. In contrast, pulsed excitation employs short, intense bursts of energy, offering advantages in time-resolved studies. Li et al. [93] utilized both CW and pulsed excitation to investigate the emission properties of hBN SPEs, reporting emission rates of 44 MHz under CW and 10 MHz under 80 MHz pulsed excitation. Notably, the purity of the SPEs under CW excitation reduced significantly as the excitation power increased, whereas purity under pulsed excitation remained high even at saturation power. Pulsed excitation also allows for time-gated correlation measurement that can improve measured single-photon purity [94].

### 2.3 Resonant versus nonresonant excitation

Resonant excitation utilizes an excitation energy that exactly matches the optical bandgap [4]. This method allows for near-deterministic excitation of the emitter, minimizing excess energy that could cause unwanted emission or phonon-induced spectral broadening. However, resonant excitation requires complicated excitation or detection schemes, such as polarization filtering [95], [96], phonon sideband (PSB) detection [97], and non-normal excitation [98], to separate the emitted photons from the excitation laser (Figure 2c and d). Wang et al. [95] employed polarization filtering to collect single photons from SPEs in InGaAs quantum dot. They reduced the polarization loss to 3.8 % instead of 50 % by coupling to polarization-selective Purcell microcavities. Extra filtering requirements sometimes limit the polarization direction and intensity. Nonresonant excitation, on the other hand, uses energy higher than the bandgap (Figure 2e). While simpler to implement, this method often results in lower-quality single photons and degrades the indistinguishability of the SPEs. Alternatively, “quasi-resonant” excitation emerged to address the above issues [99]. In this approach, two near-resonant pulses are used to predictably excite the SPEs [100], [101] (Figure 2f), allowing for natural decay or stimulated decay with a second pulse [102]. Jayakumar et al. [99] utilized a two-photon excitation scheme on InAs/GaAs QDs embedded into a microcavity. It allows for the deterministic generation of photon pairs, making the scheme suitable for generating time-bin entanglement.

## 3 Characterization of single-photon emission

**Figure 3:**
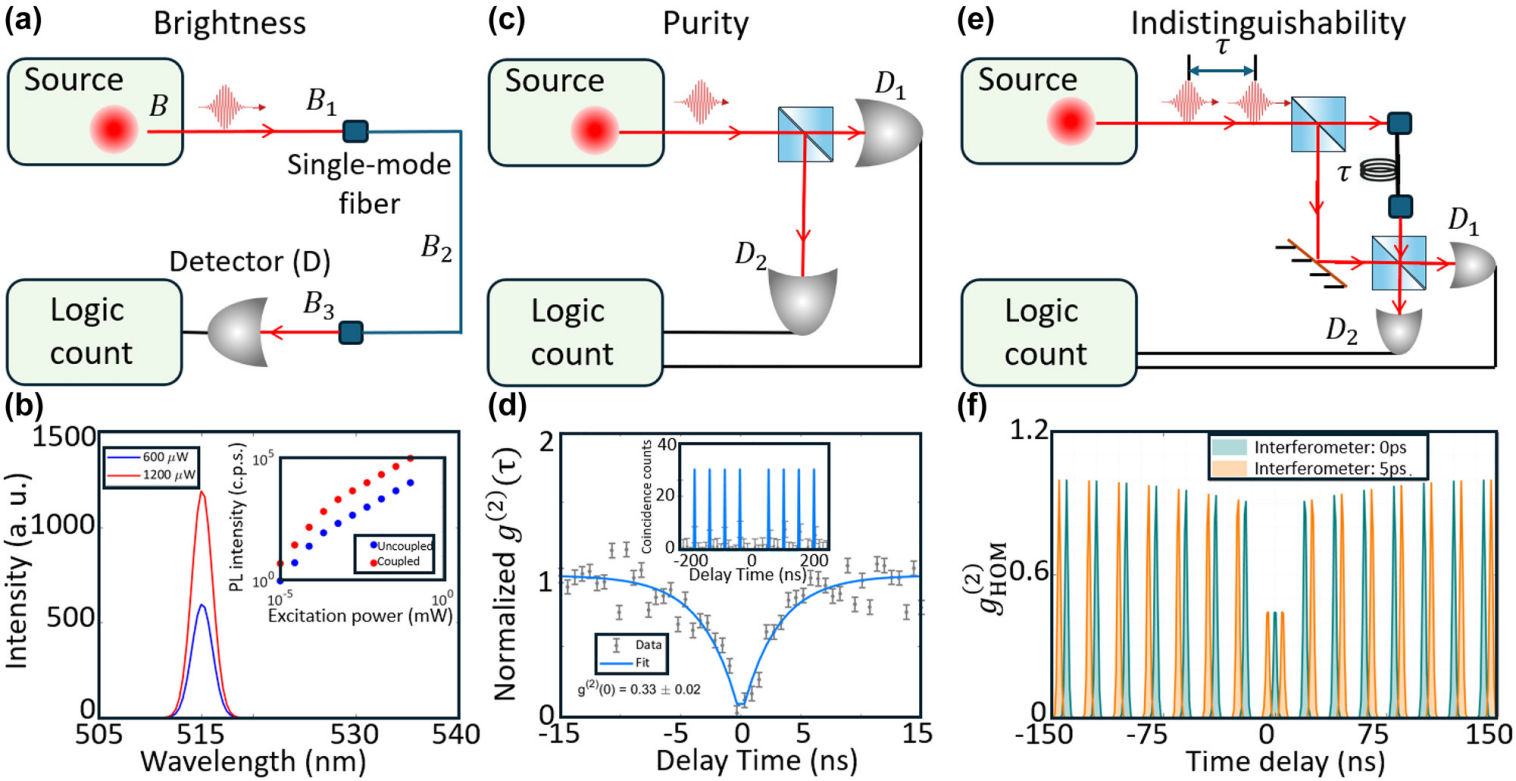
Criteria of SPEs, brightness, purity, and indistinguishability. (a) Experiment set up used to measure brightness ***B*** of an SPE under pulsed excitation conditions. The emitted photons are collected through a single-mode fiber and detected by a detector (*D*), with the brightness measured at different points (*B*_1_, *B*_2_, *B*_3_) depending on the collection efficiency. The logic count system is used to record photon detection events. (b) Brightness measurement results showing the integrated PL intensity of an SPE at two excitation powers, 600 μW (blue) and 1,200 μW (red), with emission peaks around 515 nm. The inset displays the pump-power-dependent PL intensity of a hBN SPE before and after coupled to a metallo-dielectric antenna. (c) HBT experiment setup used to measure purity of an SPE under pulsed excitation conditions. Emitted photons from the source are split by a beam splitter and directed to two detectors, *D*_1_ and *D*_2_, with the photon coincidences recorded by the logic count system. (d) Single-photon purity measured under continuous wave excitation. The data (points) and the fit (blue line) yield $g^{\left(2\right)}\left(0\right)=$ 0.33 ± 0.02. The inset shows the single-photon purity measured under pulsed excitation, demonstrating clear photon antibunching behavior with well-separated peaks in the coincidence counts. (e) HOM experiment setup used to measure indistinguishability of an SPE under pulsed excitation conditions. Two consecutive photons, separated by a time delay *τ*, are sent through a beam splitter, and their interference is measured at detectors *D*_1_ and *D*_2_. (f) Indistinguishability measurement results using the HOM effect, showing the $g_{\text{HOM}}^{\left(2\right)}\left(0\right)$ as a function of time delay for two interferometer settings: 0 ps (dark green) and 5 ps (orange). The reduction in photon correlations at zero-time delay demonstrates two-photon interference. Reproduced with permission from: (a), (c), (e), ref. [11], John Wiley and Sons; (f), ref. [103], Springer Nature.

SPEs are characterized by BPI values (Brightness, Purity, and Indistinguishability). Brightness (***B***) is quantified by PL intensity, which represents the number of collected photons per second. ***B*** is proportional to the excitation rate, quantum yield (QY), and collection efficiency. The QY quantifies the efficiency of photon emission in response to excitation, which is calculated from the number of emitted single photons at saturation normalized to the laser repetition rate. The measured value of ***B*** can vary depending on the measurement location: at the first collection element (*B*_1_), coupled inside a single mode fiber or optical path (*B*_2_), and at the detector (*B*_3_) [11]. Figure 3a shows a simple illustration of a fiber-coupled measurement scheme. ***B*** is wavelength dependent (Figure 3b) and is a function of excitation power (inset Figure 3b). The inset of Figure 3b shows the PL intensity of a defect in hBN nanoflakes before and after coupled to a metallo-dielectric antenna [93], which saturates at high power due to the finite availability of excited states and competition with nonradiative recombination processes, such as Auger recombination.

Single-photon purity (***P***) is quantified by the second order correlation function $g^{\left(2\right)}\left(\tau \right)$ , which is given by

$$g^{\left(2\right)}\left(\tau \right)=\frac{\left\langle n_{1}\left(t\right)n_{2}\left(t+\tau \right)\right\rangle }{\left\langle n_{1}\left(t\right)\right\rangle \left\langle n_{2}\left(t+\tau \right)\right\rangle },$$

where $n_{i}\left(t\right)$ is the number of counts registered by the detector *i* at time *t*, *τ* is delay time between photon detection events in two detectors, and the <…> is the time average operator [104]. $g^{\left(2\right)}\left(\tau \right)$ measures the probability that the source produces at most one photon per excitation event. Sometimes, different forms of $g^{\left(2\right)}\left(\tau \right)$ , such as $1-g^{\left(2\right)}\left(0\right)$ or the Mandel *Q* function [105] $Q\left(\tau \right)=g^{\left(2\right)}\left(\tau \right)-1$ , may be used to quantify ***P***. Figure 3c shows a simple experimental Hanbury–Brown and Twiss (HBT) setup [106], which can be used to approximate $g^{\left(2\right)}\left(\tau \right)$ in the limit where the probability of measuring more than one photon on one detector at a time is much smaller than the probability of a single-photon detection event. Photons from a light source are split by a 50:50 beam splitter and directed to two detectors, and the time correlation between detection events is measured to assess the statistical properties of the photon stream. An ideal *n*-photon Fock state exhibits $g^{\left(2\right)}\left(0\right)=1-\frac{1}{n}$ , and an ideal single-photon emitter, therefore, exhibits $g^{\left(2\right)}\left(0\right)=0$ with $g^{\left(2\right)}\left(0\right)<g^{\left(2\right)}\left(\tau \right)\forall \tau$ . When more than one emitter with identical brightness is excited simultaneously, $g^{\left(2\right)}\left(0\right)>0.5$ , although $0<g^{\left(2\right)}\left(0\right)<0.5$ is often observed when a single SPE is excited together with other weak SPEs and background fluorescence from the host material.

Figure 3d shows $g^{\left(2\right)}\left(\tau \right)$ for a 36 nm thick GaSe crystal under CW excitation [79], characterized by an exponential dip in coincidence counts at zero-time delay. This antibunching dip is fitted by $g^{\left(2\right)}\left(\tau \right)=A\text{exp}\left(-\frac{\tau }{\tau_{0}}\right)$ , where *τ*_0_ is a time constant determined by the emission lifetime *τ*_*e*_ and pumping time *τ*_*p*_ following $\frac{1}{\tau_{0}}=\frac{1}{\tau_{e}}+\frac{1}{\tau_{p}}$ . The inset of Figure 3d shows $g^{\left(2\right)}\left(\tau \right)$ of color centers in a hBN flake [107] coupled to a microcavity under pulsed excitation, with a series of peaks separated by the reciprocal of the excitation laser repetition rate, and suppressed coincidences at zero-time delay.

Indistinguishability (***I***) is quantified by *V*_HOM_, the visibility in the Hong–Ou–Mandel (HOM) experiment [108], [109]. Figure 3e illustrates a HOM experiment, where single photons are split into two paths by the first beam splitter with a delay time *τ* introduced between the paths. At the second beam splitter, quantum interference occurs, and if the photons are indistinguishable, they will exit together, leading to reduced coincidence counts for *τ* = 0. By polarization filtering or adjusting *τ*, we can obtain *V*_HOM_ [109], given by $V_{\text{HOM}}=\mathrm{Tr}\left(\hat{\rho_{1}}\hat{\rho_{2}}\right)=\frac{\mathrm{Tr}\left(\hat{\rho_{1}^{2}}\right)\;+\;\mathrm{Tr}\left(\hat{\rho_{2}^{2}}\right)\;-\;\text{O}\left(\hat{\rho_{1}},\hat{\rho_{2}}\right)}{2}$ , where $O\left(\hat{\rho_{1}},\hat{\rho_{2}}\right)=|\hat{\rho_{1}}-\hat{\rho_{2}}{|}^{2}$ is the operational distance between the states of the two photons $\hat{\rho_{1}}$ and $\hat{\rho_{2}}$ . The ***I*** is related to the optical coherence time *T*_2_ and spontaneous emission time *T*_1_ of SPEs by the approximate relation *I* = *T*_2_/2*T*_1_. The pure dephasing rate *γ* represents the rate at which a quantum system loses its coherence due to environmental interactions without energy dissipation, following $\frac{1}{T_{2}}=\frac{1}{2T_{1}}+\gamma$ . For ideal SPEs without dephasing, *T*_2_/2*T*_1_ = 1. In practice, this ratio is smaller than one because *T*_2_ is suppressed by interactions between SPEs and their environment, such as charge and spin noise [110] and phonon scattering [111]. *T*_2_ can be increased by materials engineering, while *T*_1_ can be reduced by Purcell enhancement achieved through local control of electromagnetic fields. Utzat et al. [112] demonstrated that halide perovskite quantum dots (PQDs) CsPbBr_3_ exhibit efficient single-photon emission with *T*_2_ ≈ 80 ps and *T*_1_ ≈ 210 ps at 4 K, making PQDs attractive in realizing high ***I*** SPEs among colloidal quantum dots (CQDs). Figure 3f shows the $g_{\text{HOM}}^{\left(2\right)}\left(\tau \right)$ of nanotube defects (NTDs) in sp^3^-functionalized SWCNTs coupled with an optical cavity [103], leading to a *V*_HOM_ up to 0.65 and a 217-fold enhancement in visibility.

The BPI values influence the design rules for SPEs. Table 1 demonstrates the performance of SPEs in quantum applications, where source efficiency refers to the probability of collecting a photon in each excitation pulse (proportional to ***B***). In QKD protocols, such as BB84 [128], both ***P*** and ***B*** contribute to improving the secure key rate. In other applications, such as Greenberger–Horne–Zeilinger (GHZ) state generation [125], [126], [127], all BPI values are crucial for enhancing fidelities and overall efficiencies. Other factors also affect the design rules of SPEs. For example, the emission wavelength determines the transmission properties in various media [17] (Figure 1a). SPEs with telecommunication band emission minimize the transmission losses in optical fibers, enable long-distance QKD [113], and are promising for quantum internet construction. Emission in the ultraviolet range is ideal for free-space transmission. SPEs with electrical excitation capability are ideal for integration into Complementary Metal-Oxide-Semiconductor (CMOS) circuits [34], [129]. SPEs that can emit photons at room temperature or higher [130] reduce the energy cost associated with cooling systems. SPEs that emit in a Gaussian mode facilitate seamless waveguide coupling [131].

**Table 1:** Performance of LD-SPEs in various quantum applications.

	Purity *g* ^(2)^(0)	Indistinguishability	Source efficiency
Quantum key distribution [113], [114], [115]	∼0.5 %	–	∼5 %
Boson sampling [116], [117], [118], [119]	∼2 %	∼95 %	∼55 %
Quantum teleportation [120], [121]	∼18 %	∼65 %	∼15 %
Generate cluster state [122], [123], [124]	∼5 %	∼95 %	∼18 %
Generate GHZ state [125], [126], [127]	∼2 %	∼95 %	∼30 %

In Sections 4–7, we review the progress of LD-SPEs categorized by their materials and emission wavelengths (Figure 1a). We discuss (1) semiconductor QD SPEs with emission wavelengths spanning approximately from 280 nm to 1,550 nm, (2) nanotube SPEs from 570 nm to 2,000 nm, (3) hBN SPEs from 300 nm to 850 nm, and (4) TMDCs from 600 nm to 1,550 nm. We focus in particular on SPEs capable of emitting single photons at telecommunication wavelengths, based on materials such as InAs/InP QDs, SWCNTs, and MoTe_2_.

## 4 Semiconductor quantum dots

LD-SPEs have been demonstrated in CQDs [132], graphene QDs (GQDs) [33], [133], and epitaxially grown QDs (EQDs) [17]. Single-photon emission in QDs originates from excitons formed within discrete energy levels due to quantum confinement.

CQDs are semiconductor nanocrystals with core sizes typically ranging from 2 to 10 nm, synthesized in a colloidal solution [132]. CQD SPEs are attractive due to their flexibility in synthesis [134], ease of integration, and ability to operate at room temperature [135]. CQD SPEs offer tunable emission wavelengths, which can be controlled by adjusting their size, morphology, and structure [132], [136]. Krishnamurthy et al. [137] demonstrated PbS/CdS SPEs with tunable emission wavelengths covering the telecom S band (1,460–1,530 nm) and O band (1,260–1,360 nm) at room temperature. The tunability was achieved by adjusting the core size and shell thickness of PbS/CdS CQDs. Chandrasekaran et al. [135] demonstrated near-blinking free, high purity ( $g^{\left(2\right)}\left(0\right)=0.03$ ) InP/ZnSe core/shell QD SPEs at 629 nm by leveraging tris(diethylamino)phosphine as the phosphorus precursor. The SPEs exhibited sharp room-temperature spectra and photostability. CQD SPEs in general face challenges such as low PL stability, often manifesting as blinking or intermittent fluorescence, due to strong Auger recombination.

**Figure 4:**
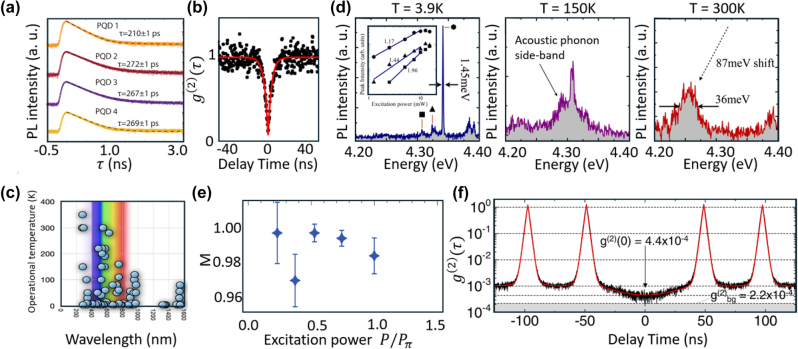
Single-photon emitters based on quantum dots. (a) The PL decay of a single PQD. The emission exhibits an initial fast decay (∼210–280 ps), followed by a slower mono-exponential decay. (b) Single-photon purity of GQD SPEs under nonresonant excitation at room temperature, yielding *g* ^(2)^(0) equals to 0.05 ± 0.05. (c) Summary plot showing emission wavelengths and operational temperatures of various EQD SPEs. (d) PL spectra of EQD SPEs measured at different temperatures: 3.9 K (left), 150 K (middle), and 300 K (right). At 3.9 K, the inset shows a power dependence plot with a fitted slope. At 150 K, the spectrum displays an acoustic phonon sideband. At 300 K, the PL peak exhibits a shift of 87 meV. (e) Indistinguishability M of InGaAs SPEs as a function of excitation power, measured at 4.2 K. The indistinguishability reaches a maximum value of 0.9956. Error bars are based on Poissonian statistics from detected events. (f) The purity of an InAs/InP QD in an optical horn at 8 K under quasi-resonant excitation with a $g^{\left(2\right)}\left(0\right)$ value of 4.4 × 10^−4^ and a background correction value of 2.2 × 10^−4^. Adapted with permission from: (a), ref. [112], The American Association for the Advancement of Science; (b), ref. [33], Springer Nature; (c), ref. [17], AIP Publishing; (d), ref. [16], Copyright 2014, American Chemical Society; (e), ref. [147], Springer Nature; (f), ref. [148], AIP Publishing.

As a specific type of CQD, halide perovskites QDs (PQDs) exhibit room-temperature single-photon emission, near-unity QY, and high photostability. PQDs are synthesized through methods [138] such as hot injection [139], [140], ligand-assisted precipitation [141], [142], and ultrasonic synthesis [143], [144]. Liu et al. [145] demonstrated 100 % QY in CsPbI_3_ PQDs at room temperature, employing a synthetic protocol involving the introduction of an organometallic compound, trioctylphosphine-PbI_2_, as the reactive precursor. Tang et al. [146] demonstrated CsPbBr_3_/CdS core/shell PQDs with nonblinking PL and a high QY of 90 %, attributed to the reduction of electronic traps within the stable core/shell structure. Utzat et al. [112] showed that CsPbBr_3_ SPEs exhibit fast emission lifetimes of 210–280 ps (Figure 4a) with a large *T*_2_/2*T*_1_ ratio (∼0.2) and stable emission over several minutes at cryogenic temperatures at 520 nm (2.38 eV). Zhu et al. [149] reported CsPbI_3_ SPEs with 98 % ***P*** ( $g^{\left(2\right)}\left(0\right)=0.02$ ) at room temperature, demonstrating that enhanced quantum confinement is key to improving purity by suppressing biexciton emission. Kaplan et al. [150] measured the $g_{\text{HOM}}^{\left(2\right)}\left(\tau \right)$ of single photons from CsPbBr_3_ CQDs at 3.9 K, showing corrected visibilities up to 0.56 ± 0.12 without using any cavity. PQDs exhibit tunable wavelengths ranging from 400 nm to 800 nm that can be controlled by adjusting their composition, size, morphology, and dimensions. Jun et al. [151] coupled CsPbBr_3_ nanocrystals to circular Bragg grating cavities, exhibiting 5.4-fold PL enhancement with lifetimes reduced to less than 100 ps. CsPbI_3_ PQDs integrated into optical microcavities have also been shown to exhibit narrow room-temperature linewidths (∼1 nm) [152].

CQD SPEs face several bottlenecks waiting to be addressed: strong Auger recombination reduces the PL intensity; intense multiexciton emission restricts HOM experiments to cryogenic temperatures; blinking leads to poor photostability at ambient conditions; and there are no reports to date of telecommunications band operation.

Graphene QDs (GQDs) are atomically thin fragments of graphene, typically consisting of 1 or 2 layers with lateral sizes below 10 nm [153]. Compared to graphene, GQDs exhibit desirable properties for SPEs, such as a bandgap opened as a result of quantum confinement and tunable physical properties enabled by geometry engineering and chemical functionalization. GQD SPEs are synthesized by bottom-up methods, such as molecular fusion, allowing control over size, morphology, doping, functionalization, and synthesis techniques. Zhao et al. [33] demonstrated GQD SPEs at room temperature with high ***B***, ***P*** ( $g^{\left(2\right)}\left(0\right)=0.05$ ) and no blinking (Figure 4b). The emission wavelengths were tunable from ∼650 nm to ∼750 nm by chlorine functionalization. Theoretical studies indicate that amino-group-functionalized GQDs exhibit single-photon emission [154]. GQD SPEs commonly suffer from low ***I*** [154]. Coupling GQD SPEs to photonic cavities remains an outstanding challenge that will be essential to the development of GQDs as technologically viable SPEs.

EQDs are synthesized through layer-by-layer techniques such as Molecular Beam Epitaxy (MBE) and Metal–Organic Chemical Vapor Deposition (MOCVD) [155]. SPEs with tailored nanostructures can be formed by tuning growth conditions, strain, and utilizing prepatterned substrates along with postgrowth techniques like etching and lithography [156], [157]. EQD SPEs cover a broad emission wavelength range, from the ultraviolet (below 280 nm) to the telecom band (around 1,550 nm) (Figure 4c). Emission in the UV range is achieved using III-nitrides, particularly GaN/AlGaN EQDs. Holmes et al. [16] demonstrated that GaN/AlGaN SPEs maintain high ***P*** ( $g^{\left(2\right)}\left(0\right)=0.34$ ) at temperatures up to 350 K. Figure 4d illustrates the variation of PL intensity with temperature. The robustness is attributed to the large biexciton binding energies (∼50 meV) [158].

InGaN, InGaN/GaN, InP, and various II–VI EQDs [159], [160], such as CdSe/ZnSe, have been used to realize SPEs with emission in the visible region. Fedorych et al. [160] demonstrated CdSe/ZnSSe/MgS EQD SPEs with $g^{\left(2\right)}\left(0\right)=0.16$ under CW excitation at 300 K. Quitsch et al. [161] demonstrated electrical excitation in the same structure. Deshpande et al. [162], [163] demonstrated InGaN/GaN SPEs operating at 620 nm with $g^{\left(2\right)}\left(0\right)=0.37$ under pulsed excitation and $g^{\left(2\right)}\left(0\right)=0.32$ under CW excitation at 280 K. Cho et al. [164] realized InGaN SPEs operating at 473 nm with $g^{\left(2\right)}\left(0\right)=0.11$ at 10 K.

III-arsenide materials, such as InAs/GaAs EQDs, have been used to achieve single-photon emission in the near-infrared (NIR) region with near-unity ***I***. He et al. [165] demonstrated InAs/GaAs SPEs operating at a wavelength of 940 nm with $g^{\left(2\right)}\left(0\right)=0.012$ and ∼97 % HOM visibility at 4.2 K. The combined purity and indistinguishability of this source allowed for the realization of a quantum CNOT gate suitable for generating entangled states from the SPE. Somaschi et al. [147] demonstrated InGaAs SPEs operating at a wavelength of 890 nm with $g^{\left(2\right)}\left(0\right)=0.0028$ and 99.56 % visibility under resonant excitation at 4.2 K (Figure 4e), along with ***B*** more than an order of magnitude higher than SPDC sources. Wang et al. [116] demonstrated 3-, 4-, and 5-photon boson sampling with sampling rates of 4.96 kHz, 151 Hz, and 4 Hz, respectively. This application leveraged the high ***P*** ( $g^{\left(2\right)}\left(0\right)$ = 0.027) and ***I*** (0.900) of InAs/GaAs EQD SPEs integrated with a micropillar cavity and low-loss photonic circuits. Schweickert et al. [166] achieved $g^{\left(2\right)}\left(0\right)=\left(7.5\pm 1.6\right)\times 10^{-5}$ at a wavelength of 790 nm by utilizing two-photon excitation of the biexciton state in a GaAs/AlGaAs quantum dot at 4 K.

The telecommunication band is primarily covered by InAs/InP and InAs/GaAs EQDs due to their narrow bandgaps (0.2 eV–1.2 eV). The emission wavelengths of InAs/InP SPEs can be tuned to 1,550 nm (telecom C-band) [167]. Takemoto et al. [113] demonstrated InAs/InP SPEs with high purities ( $g^{\left(2\right)}\left(0\right)=0.002$ after background correction) by employing an optical horn structure [168] to enhance photon extraction efficiency and demonstrated QKD over 120 km. Miyazawa et al. [148] demonstrated InAs/InP SPEs operating at 1,500 nm with $g^{\left(2\right)}\left(0\right)=4.4\times 10^{-4}$ at 8 K (Figure 4f). Muller et al. [169] showed that InAs/GaAs EQD SPEs exhibit antibunching at 1,550 nm with $g^{\left(2\right)}\left(0\right)=0.11$ at 4 K, and they leveraged a biexciton cascade mechanism to generate entangled photons with entanglement fidelity of 87 % that remained stable at temperatures up to 94 K. Nawrath et al. [170] demonstrated InAs/InGaAs/GaAs SPEs operating at 1,550 nm with $g^{\left(2\right)}\left(0\right)=0.072$ under near-resonant excitation at 4 K.

EQD SPEs demonstrated room-temperature emission in the UV or visible range, with tunable emission wavelengths controlled by adjusting the material’s stoichiometry during growth [17]. Thanks to the maturity of EQD growth techniques, these SPEs exhibit promising scalability for quantum applications such as boson sampling [116], [117], [118], linear cluster state generation [122], [123], [124], QKD [113], [114], quantum logic gate operation [171], and quantum teleportation [120], [121]. While promising, EQD SPEs face several challenges: HOM measurements are only favorable at cryogenic temperatures due to strong dephasing rates at room temperature; it remains challenging to achieve usable photon indistinguishability for photons emitted from distinct EQD SPEs; and the difficulty in achieving electrical excitation limits the potential for integration into on-chip devices.

## 5 Nanotubes

1D nanotubes SPEs are primarily realized using two materials: boron nitride nanotubes (BNNTs) and SWCNTs. BNNTs, structurally similar to rolled boron nitride sheets, are known for their wide bandgap and high thermal and chemical stability [172]. BNNT SPEs exhibit room-temperature single-photon emission in the 570–610 nm range [173], [174], [175]. Chejanovsky et al. [173] reported SPEs in BNNT with $g^{\left(2\right)}\left(0\right)=0.33$ , demonstrating single-photon emission in hybrid/entwined BNNTs. Ahn et al. [174] studied point defects in 50-nm-diameter BNNTs, demonstrating $g^{\left(2\right)}\left(0\right)=0.38$ under nonresonant excitation at room temperature, with spectral modulation enabled via a NIR control laser. Gao et al. [175] observed spin defects in BNNTs with a spin *S* = 1/2 ground state and no intrinsic quantization axis, in BNNTs, exhibiting $g^{\left(2\right)}\left(0\right)<0.5$ under nonresonant excitation at room temperature. These spin defects have potential for magnetic sensing, with a typical DC magnetic field sensitivity of ∼80 μT/ $\sqrt{\text{Hz}}$ .

**Figure 5:**
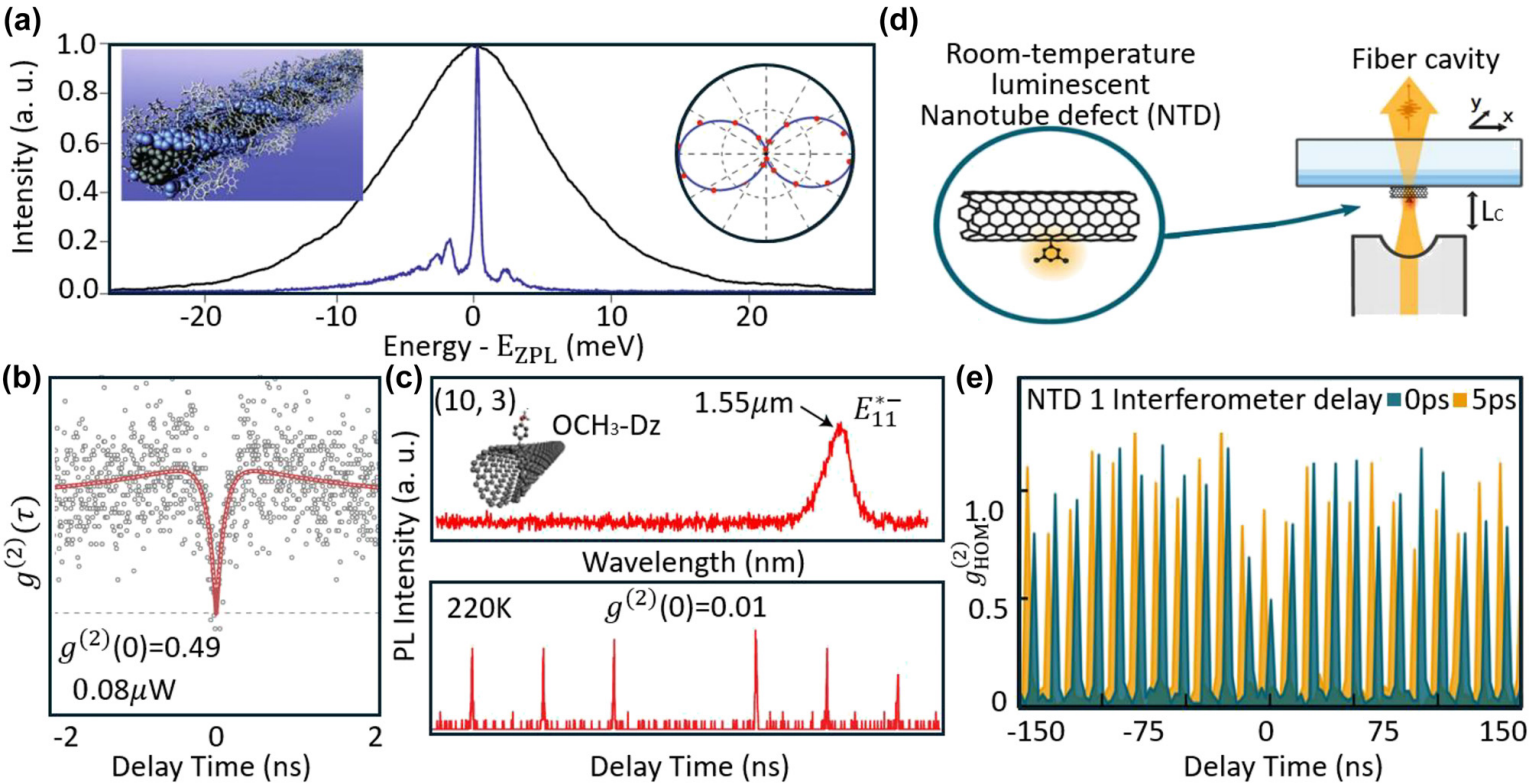
Single-photon emitters based on SWCNTs. (a) PL spectrum of a single SWCNT at room temperature (black line) and at 10 K (blue line) [176]. Inset: a polymer wrapped nanotube leading to reduced spectral diffusion and blinking [177] (left); PL polarization diagram (right). (b) Purity of electrically excited SWCNT SPEs at 1.6 K, yielding *g* ^(2)^(0) equals to 0.49 at 0.08 μW excitation power. (c) PL spectrum and purity for SPEs in (10, 3) SWCNTs functionalized with OCH_3_-Dz. The PL spectrum shows an emission peak at 1.55 μm corresponding to the *E*_11_^∗^ transition. The $g^{\left(2\right)}\left(0\right)$ equals to 0.01 measured at 220 K demonstrates high purity of the single-photon emission. (d) Schematic of nanotube defect (NTD) SPEs operating at room temperature and coupled to a tunable fiber cavity. The fiber cavity setup allows precise control of the cavity length (*L*_C_) and enhances emission properties of the NTD SPEs. (e) The HOM second-order correlation function of an NTD SPE. HOM autocorrelation function of an NTD, measured in a copolarized interferometer configuration with interferometer delays of 0 ps (dark green) and 5 ps (orange). The zero-interferometer delay corresponds to a delay equal to the separation of one excitation pulse. The visibility is then measured to be *v* = 0.65 ± 0.24 at room temperature. Adapted with permission from: (a), ref. [176], Springer Nature; (b), ref. [178], Springer Nature; (c), ref. [74], Springer Nature; (d), (e), ref. [103], Springer Nature.

SWCNTs consist of covalently bonded carbon atoms arranged in an ordered tubular structure, with their diameter and roll-up angle defined by the chiral index (***n***, ***m***) where ***n*** and ***m*** specify the wrapping direction of the graphene lattice. The chiral index also defines the emission wavelength for intrinsic SWCNTs. SWCNTs stand out as candidates for SPEs due to their structure-specific NIR PL [176] (Figure 5a). Single-photon emission from SWCNTs originates from excitons confined in potential wells created through noncovalent or covalent functionalization.

Noncovalent functionalization creates localized potential wells through unintentional molecular adsorption or local inhomogeneities at the interface with the surrounding matrix or substrate [179], allowing control of exciton diffusion and inducing strong photon antibunching. This approach leverages the sensitivity of SWCNTs to their dielectric environment while preserving their excellent optical characteristics [180]. Högele et al. [181] demonstrated strong photon antibunching in CoMoCat SWCNTs encapsulated in sodium dodecylbenzenesulfonate, achieving $g^{\left(2\right)}\left(0\right)=0.03$ at 10 K. The SPEs maintained high ***P*** while tuning the emission wavelength between 855 and 885 nm by adjusting the temperature from 4.2 K to 25 K. Khasminskaya et al. [178] demonstrated electrically excited SWCNT SPEs at 1.6 K by integrating HiPco SWCNTs with a waveguide circuit. The ***P*** ( $g^{\left(2\right)}\left(0\right)=0.49$ ) of these SPEs was limited by the timing resolution of the detector (Figure 5b). Raynaud et al. [179] quantified potential energy disorder along the tube axis using hyperspectral imaging and quasi-resonant excitation spectroscopy, revealing that interface roughness leads to exciton localization at low temperatures, resulting in segmented photoluminescence lines and random potential traps with a 70 nm spacing and 20 meV energy spread. However, noncovalent functionalization persists its weak interaction with SWCNTs only at cryogenic temperature, making the fabricated SPEs unsuitable for room-temperature applications.

Covalent functionalization has enabled a variety of approaches for creating localized excitons through methods such as oxygen doping, diazonium salts, DNA, or photoexcited aromatics-based functionalization. Ma et al. [59] demonstrated solitary oxygen dopant SWCNT SPEs with $g^{\left(2\right)}\left(0\right)=0.32$ at 298 K in the 1,100–1,300 nm wavelength range, achieved by incorporating undoped (6,5) SWCNTs into a SiO_2_ matrix. Zheng et al. [182] reported single-photon emission from coupled defect states in DNA-functionalized SWCNTs, with $g^{\left(2\right)}\left(0\right)=0.27$ at room temperature, where guanine defects in ssDNA strands created multiple coupled trapping sites due to dense covalent functionalization.

Aryl sp^3^ defects created through diazonium-based reactions have emerged as promising candidates for SPEs, offering stable, shot-noise limited emission. These defects are synthetically tunable, allowing for enhanced trapping potentials and red-shifted emission, particularly in large-diameter tubes emitting at telecom wavelengths. He et al. [74] demonstrated stable SPEs with $g^{\left(2\right)}\left(0\right)=0.01$ and telecom wavelength (1,550 nm) emission from SWCNT sp^3^ defects at room temperature. They explored the room-temperature PL of SWCNTs with different chiral indices, functionalized with Cl_2_-Dz and MeO-Dz. By using DOC and PFO-bpy coatings, they achieved emission in the 1,300–1,550 nm range for (6,5), (7,5), and (10,3) SWCNTs (Figure 5c). While aryl sp^3^ defect SPEs typically exhibit low *T*_2_/2*T*_1_ ratios (around 1 %), this can be improved by coupling to optical cavities [176]. Husel et al. [103] conducted HOM experiments on individual NTDs coupled to an optical microcavity (Figure 5d), achieving a visibility of 0.65 and $g^{\left(2\right)}\left(0\right)=0.31$ at room temperature in the telecom band. This was the first room-temperature demonstration of cavity-enhanced ***I*** for LD-SPEs, despite the high dephasing rate (Figure 5e). Unfortunately, covalently functionalized SWCNT SPEs suffer from low QY (10–30 %) due to the strong nonradiative decay of excitons at defects [176], [183] and are prone to spectral diffusion and blinking [176]. Further, the ***B*** of SWCNT SPEs is limited by strong exciton–exciton annihilation (EEA) arising from their one-dimensional nature. These limitations hinder the use of SWCNT SPEs in quantum applications. Advances in defect engineering and cavity coupling offer potential solutions to these challenges.

## 6 Few-layer hexagonal boron nitride

**Figure 6:**
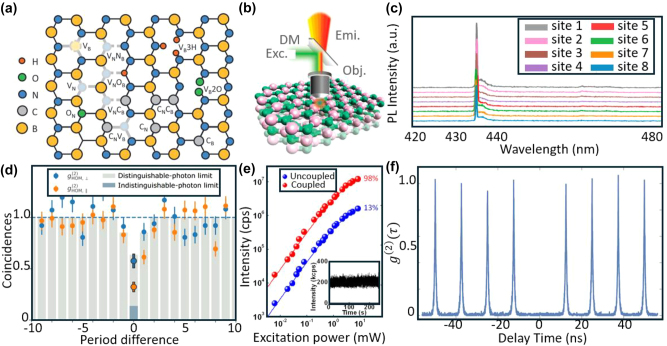
Structure, defects, and performance of hBN SPEs. (a) Schematic of a hBN lattice structure highlighting various types of defects it can host. The lattice is composed of boron (B, yellow) and nitrogen (N, blue) atoms. Common defects include nitrogen vacancies (V_N_), boron vacancies (V_B_), oxygen substituting nitrogen or boron (O_N_, O_B_), carbon substituting nitrogen or boron (C_N_, C_B_), and complex vacancies with multiple atoms missing or substituted (e.g., V_B_3H, V_B_2O). (b) Schematic demonstrating the separation of excitation and emission light using a dichroic mirror (DM) during the optical characterization of hBN. The excitation (Exc.) light is directed onto the sample via an objective (Obj.), while the emitted (Emi.) light is reflected by the DM and collected for analysis. (c) Low-temperature spectra of the eight hBN SPEs, labeled 1 through 8. The ZPL for all emitters is reproducible, centered around 436 ± 0.7 nm. (d) Two photon interference between successively emitted photons from the same source with a delay of 12.5 ns, yielding a *V*_HOM_ of 0.56 ± 0.11. (e) PL intensity versus excitation power for hBN SPEs, comparing uncoupled (blue) and coupled (red) configurations using a metallo-dielectric antenna setup. The coupled system achieves a near-unity photon collection efficiency of 98 %, compared to 13 % for the uncoupled case. The inset shows the emitter intensity over time, demonstrating excellent temporal stability without blinking. (f) High purity hBN SPEs with $g^{\left(2\right)}\left(0\right)$ equals to 0.0064 under pulsed excitation. Adapted with permission from: (a), ref. [46], AIP Publishing; (b), ref. [67], Copyright 2016, American Chemical Society; (c), ref. [185], Springer Nature; (d), ref. [186], American Physical Society; (e), ref. [93], Copyright 2019, American Chemical Society; (f), ref. [107], American Physical Society.

Two-dimensional hBN is a wide-bandgap insulator (*E*_g_ ∼ 5.97 eV) with a graphene-like honeycomb lattice of alternating boron and nitrogen atoms. The wide bandgap makes hBN SPEs robust against thermal fluctuations, enabling stable single-photon emission at room-temperature. Few-layer hBN SPEs show promising characteristics such as high BPI values and spin-photon interfaces. hBN SPEs have been successfully integrated with photonic circuits in initial demonstrations of scalable quantum photonic technologies [38], [39]. Single-photon emission in hBN arises from trapped excitons at defect sites, including nitrogen vacancies (V_N_), boron vacancies (V_B_), antisite carbon vacancies (V_N_C_B_), and antisite nitrogen vacancies (V_N_N_B_) [184] (Figure 6a), which can be introduced by annealing [67], [187], electron beam [185], [188], [189] or ion beam [69], [189] irradiation, nanopillars [190], [191], plasma processing [72], [73], or femtosecond pulses [68], [192]. Theoretical studies [193], [194], [195], [196] have proposed new type of defects, such as C_2_C_N_ and C_2_C_B_ carbon clusters [197], [198], as candidates for SPEs in hBN. hBN SPEs exhibit zero phonon lines (ZPLs) across the NIR-visible range (∼560–780 nm) [67], [80] and the UV range (∼300 nm) [87], [199].

Tran et al. [53], [54] first demonstrated single-photon emission from monolayer and multilayer hBN SPEs, with stable emission at 623 nm over 10 min and $g^{\left(2\right)}\left(0\right)<0.5$ . Figure 6b illustrates the optical characterization method they used, where excitation and emission are separated by a dichroic mirror during measurements. Bourrellier et al. [87] demonstrated UV hBN SPEs using electron beam irradiation, achieving operation at 300 nm with postprocessed $g^{\left(2\right)}\left(0\right)=0.2$ at room temperature. Previously, this emission wavelength was only achieved by III-nitride EQD SPEs.

Compared with many other platforms, hBN SPEs show reproducible emission wavelengths. Fournier et al. [185] demonstrated hBN SPEs operating at 436 nm that were created at controlled locations using electron beam irradiation. The local irradiation process activates SPE ensembles with submicron precision, leading to ZPLs consistently centered at 436 ± 1 nm (Figure 6c). Horder et al. [200] employed resonant excitation to characterize the emission line shape, demonstrating coherence of optical transitions through the observation of Rabi oscillations. Fournier et al. [186] conducted HOM experiments on the blue hBN SPEs, demonstrating corrected HOM visibility of 0.56 ± 0.11 at cryogenic temperatures (Figure 6d). The blue hBN SPEs exhibit spectral stability, room-temperature operation, ultra-narrow linewidth, and high ***I*** under nonresonant excitation, making them favorable for quantum frequency conversion (QFC) to telecommunications wavelengths.

Li et al. [93] demonstrated hBN SPEs coupled to metal-dielectric antennas, achieving near-unity light collection efficiency (98 %) and a QY of 12 % (Figure 6e). The SPEs exhibited high ***B*** (10^7^ cps at 10^1^ mW excitation power) and maintained single-photon emission under excitation powers up to 8 mW at room temperature. Vogl et al. [107] demonstrated hBN SPEs integrated with a tunable microcavity [201] consisting of a hemispherical and flat mirror, achieving corrected $g^{\left(2\right)}\left(0\right)=0.0064$ at room temperature (Figure 6f). They measured single-photon interference with Michelson-type interferometers, demonstrating interferometric visibilities of up to 98.58 %.

These properties make hBN SPEs well-suited for several quantum applications. White et al. [202] demonstrated quantum random number generation (QRNG) using hBN SPEs coupled to an on-chip photonic waveguide structure at room temperature. Samaner et al. [115] integrated a hBN SPE into a QKD system based on the B92 protocol, achieving a sifted key rate of 238 bps with a quantum bit error rate of 8.95 % at a 1 MHz clock rate. Scognamiglio et al. [203] demonstrated that hBN SPEs operating at 417 nm show promise for underwater quantum communications.

**Figure 7:**
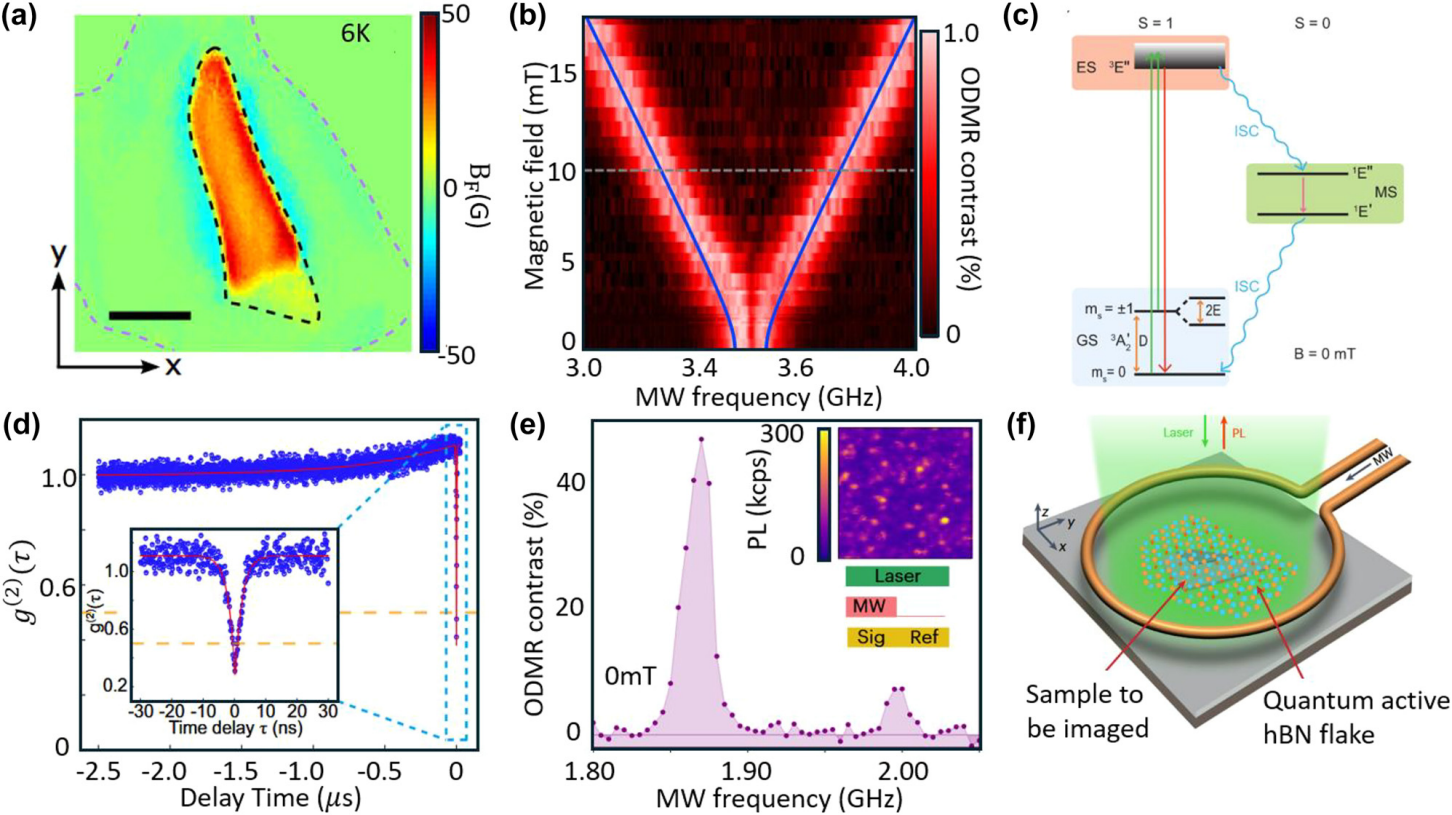
Quantum spin sensing based on hBN defects. (a) Wide-field imaging of magnetization of an exfoliated Fe_3_GeTe_2_ flake by V_B_^−^ spin defects in hBN. (b) Dependence of ODMR frequencies on the magnetic field. Experimental data (red) and fit (blue line) with parameters *D*/*h* = 3.48 GHz, *E*/*h* = 50 MHz and *g* = 2.000. (c) Simplified V_B_^−^ energy-level diagram and the transitions among the ground state (^3^A_2_′), the excited state (^3^E′), and the metastable state (^1^E′, ^1^E′′). (d) $g^{\left(2\right)}\left(\tau \right)$ of carbon-related defects in hBN. Inset indicates the fitted $g^{\left(2\right)}\left(0\right)$ = 0.25 ± 0.02. (e) The ODMR spectrum of a single defect in hBN measured in the absence of magnetic field. The top-right inset shows a confocal image of the PL intensity of the hBN device under 532 nm laser illumination. The bottom-right inset shows the pulse sequences used in the measurement. (f) A schematic illustration of quantum microscopy with spin defects in hBN. The setup includes a quantum active hBN flake and a sample to be imaged. The laser is used for excitation, and PL is collected for imaging. The microwave (MW) input enables control of the spin defects in the hBN for quantum sensing applications. Adapted with permission from: (a), ref. [211], Springer Nature; (b), ref. [207], Springer Nature; (c), ref. [208], Copyright 2021, American Chemical Society; (d), ref. [213], Springer Nature; (e), ref. [214], Springer Nature; (f), ref. [215], Springer Nature.

One of hBN SPE’s most unique properties is its compatibility with spin-based quantum sensing [204]. Optically active spin defects, such as nitrogen vacancies [205], [206], exhibit a ground-state spin that can be optically addressed and manipulated. The manipulation of the spin states through external magnetic fields [207], temperature variation [208], [209], or strain [208], [210] forms the basis for their application in quantum sensing [211], [212]. These defects have the potential to detect minute changes in environmental parameters like magnetic fields or temperature at the nanoscale, leading to applications in high-sensitivity quantum sensing devices (Figure 7a). One of the most powerful techniques for characterizing and manipulating the spin properties of defects in quantum materials is Optically Detected Magnetic Resonance (ODMR) [216]. ODMR enables the detection of spin transitions in a defect’s electronic structure by combining microwave excitation and optical readout (Figure 7b). In the case of hBN spin defects, this method involves monitoring the fluorescence from the defect centers while applying microwave radiation to induce spin transitions between different energy levels.

The first spin signature for hBN defects was observed in boron vacancies (V_B_^−^), which exhibit a broad optical emission spectrum centered at ∼800 nm [207], [210]. These defects, which exhibit a broad optical emission spectrum centered around 800 nm, are characterized by a ground-state spin triplet (*S* = 1). The spin axis aligns along the c-axis of the hBN crystal, with a zero-field splitting of *D* ≈ 3.45 GHz separating the spin |0> and spin |±1> sublevels [208], [217] (Figure 7c). Through careful laser pulsing, the V_B_^−^ defect’s spin states can be initialized, manipulated, and optically read out. However, due to their relatively low optical quantum efficiency, ODMR measurements have been limited to ensemble-level experiments rather than single-spin resolution.

In contrast, carbon-related spin defects in hBN [218], [219], some also with a spin triplet (*S* = 1), exhibit advantages for single-photon emission (Figure 7d). These defects, which emit light in the visible spectrum range, demonstrate bright room-temperature emission with more than 80 % of the emission occurring from the ZPL [220], [221]. They also show high ODMR contrast over 30 % (Figure 7e) and a long dephasing time, exceeding 100 ns [213], [222]. These features make them highly suitable for practical quantum sensing applications. Notably, these carbon-based defects achieve an estimated DC magnetic field sensitivity of approximately 3 μT/ $\sqrt{\text{Hz}}$ [214], placing them on par with the well-established NV centers in diamond, which are commonly used for magnetometry. Interestingly, some carbon-related defects show no zero-field splitting in their ODMR curves, indicating that these are spin doublets (*S* = 1/2) [219], [223], [224]. The coexistence of spin triplet and spin doublet states within a single hBN crystal matrix adds another layer of versatility to the material, making it an attractive platform for quantum sensing across a range of applications. The ability to integrate different types of spin states within a single host material opens the door to more flexible and tunable quantum devices.

Optically active spin defects in hBN bring two primary advantages over traditional NV centers in diamond, particularly for quantum sensing applications. First, quantum sensors in hBN have higher photon extraction efficiency: The 2D nature of hBN provides superior photon extraction efficiency compared to diamond. In hBN, the emission originates from the surface, minimizing internal photon losses caused by total internal reflection – a problem commonly encountered in bulk diamond. This enhanced efficiency can improve the signal-to-noise ratio in quantum measurements, making hBN a promising alternative for quantum sensing. Second, the quantum sensors in hBN could potentially offer better sensitivity and spatial resolution. Due to its atomically thin, planar structure, hBN quantum sensors can be positioned just a few ångströms away from the target object, leading to unprecedented sensitivity and spatial resolution. This capability is particularly important for applications such as imaging magnetic domains in 2D materials, where proximity to the sample is critical. The atomically smooth surface of hBN further enhances the sensing potential by reducing signal interference from surface roughness. The unique properties of hBN defects have already been demonstrated in experimental setups, including quantum microscopes [215], [225] and fiber-integrated devices [226] (Figure 7f). As research progresses, hBN is likely to play a pivotal role in the future of quantum technology, providing a versatile, high-performance platform for both fundamental studies and practical quantum devices.

Several challenges hinder the broader application of hBN SPEs. The uniformity of hBN SPEs is limited due to inherent challenges in material manipulation and control at the nanoscale that prevent the consistent creation of identical defects and often result in the formation of unintended defect types. Most hBN SPE ZPLs exist at wavelengths of 400–800 nm, with telecom wavelength emission yet to be demonstrated, limiting integration into optical fiber applications. Both the QY (current record of 12 %) [93] and ***I*** (current record of 56 %) [186] of hBN SPEs must be improved to realize technologically relevant hBN-based quantum photonic applications.

## 7 Few-layer transition metal dichalcogenides

**Figure 8:**
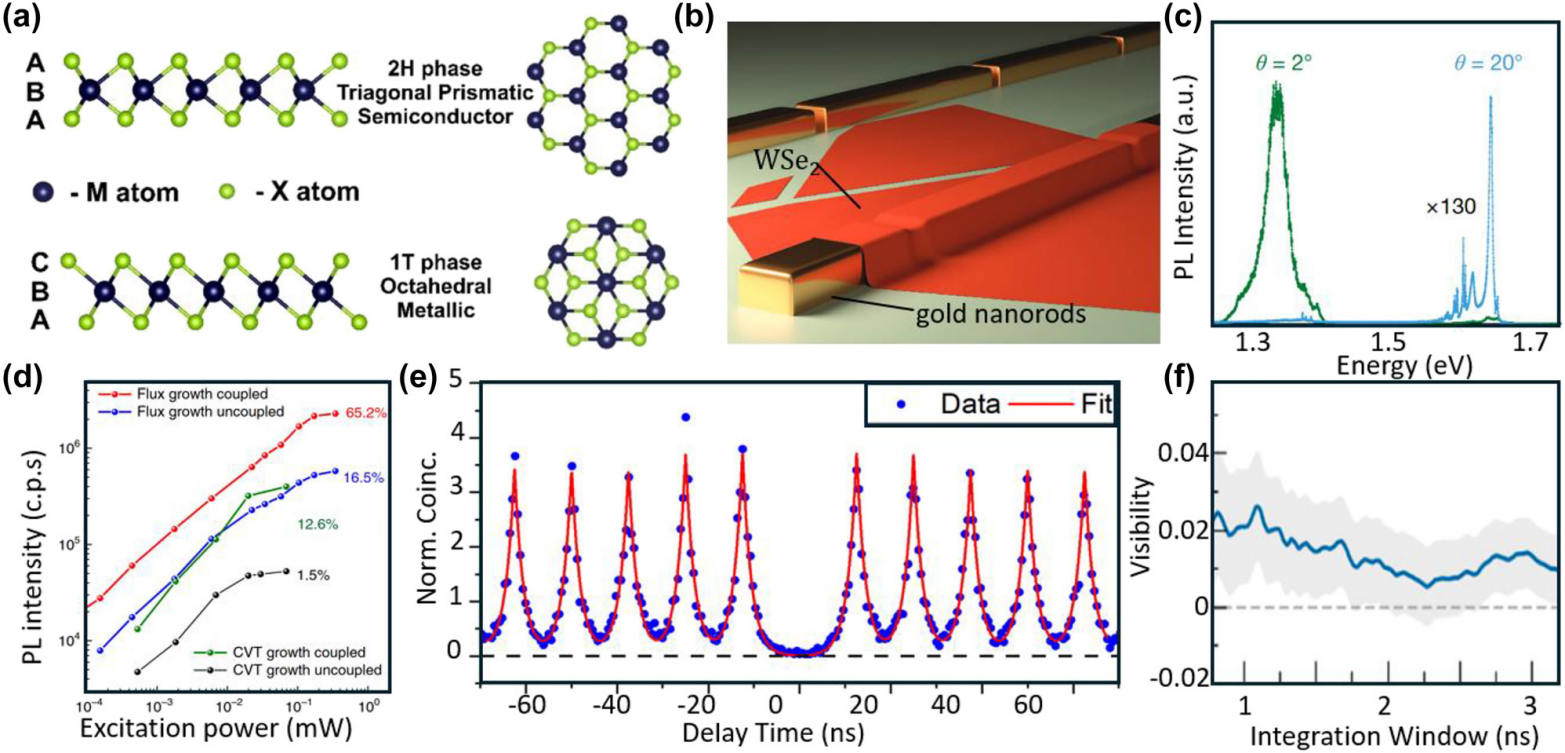
Structure, strain, and performance of layered TMDC SPEs. (a) Atomic structures of two crystallographic phases of TMDCs. The 2H phase (top) features a trigonal prismatic coordination of metal atoms (M) and chalcogen atoms (X), with an A-B-A stacking sequence. The 1T phase (bottom) is characterized by octahedral coordination and a C-B-A stacking sequence. The side and top views highlight the differences in atomic arrangements between the two phases. (b) Illustration of the WSe_2_ monolayer transferred over gold nanorods. The strain induced by folds and wrinkles formed during the transfer process, particularly over the gaps between nanorods, leads to the localization of SPEs. (c) PL spectra from MoSe_2_/WSe_2_ heterobilayers with twist angles of 2° (green) and 20° (blue; intensity scaled by 130×). The twist angle significantly impacts the PL characteristics, with the 2° sample showing a strong peak near 1.3 eV and the 20° sample exhibiting multiple peaks around 1.6 eV. (d) PL intensity of WSe_2_ SPEs grown via flux and CVT methods, both before and after coupling to an optical cavity. Flux-grown SPEs show a quantum yield of up to 65.2 % after cavity coupling (red), compared to 16.5 % without coupling (blue). CVT-grown SPEs achieve a quantum yield of 12.6 % with coupling (green) and 1.5 % without coupling (black). (e) Second-order autocorrelation function $g^{\left(2\right)}\left(\tau \right)$ for SPEs in a WSe_2_ monolayer under pulsed quasi-resonant excitation. The blue data points represent experimental measurements, while the red curve is a fit. The pronounced antibunching at zero delay time, with $g^{\left(2\right)}\left(0\right)$ equals to 0.036 ± 0.004. (f) HOM interference visibility *V*_HOM_ as a function of the temporal postselection window size for SPEs in a WSe_2_ monolayer coupled to a tunable open optical cavity. The blue line represents the measured visibility, with error bounds in gray. Visibility decreases with increasing integration window size, yielding *V*_HOM_ equals to 0.02. Adapted with permission from: (a), ref. [227], Licensee MDPI, Basel, Switzerland; (b), ref. [228], John Wiley and Sons; (c), ref. [229], Springer Nature; (d), ref. [230], Springer Nature; €, ref. [231], IOP Publishing; (f), ref. [232], American Chemical Society.

The TMDCs have a layered MX_2_ structure with hexagonally arranged X-M-X units [227] (Figure 8a). The 2D TMDCs offer strong light–matter interaction [233], [234], direct bandgaps, large exciton binding energies (0.5–1 eV) [235], [236], and valley degrees of freedom [237], allowing for circularly polarized excitonic optical transitions and efficient tuning via magnetic field. The 2D TMDC-based SPEs offer high photon extraction efficiency, ease of coupling with external fields, and seamless integration into photonic circuits [38], [39]. Single-photon emission in TMDCs originates from excitons trapped by localized strain [228] or defects [238]. Strain is introduced using bubbles [239], [240], patterned nanostructures [241], [242], nanopillars [75], [230], or atomic force microscopy (AFM) tips [243], [244] to funnel excitons into localized regions (Figure 8b). Defects are introduced by material growth [55], [56], [57], [58], electron beam [245], [246], and ion beam irradiation [240].

Over the past decade, SPEs have been demonstrated in TMDCs such as WSe_2_ [55], [56], [57], [58], WS_2_ [241], [247], MoS_2_ [248], [249], [250], MoSe_2_ [251], [252], and MoTe_2_ [38]. Palacios-Berraquero et al. [247] demonstrated electrically excited SPEs in layered WSe_2_ and WS_2_. The SPEs in WSe_2_ emitted at 760 nm with $g^{\left(2\right)}\left(0\right)=0.29$ , while the SPEs in WS_2_ emitted at 640 nm with $g^{\left(2\right)}\left(0\right)=0.31$ . The SPEs demonstrated seamless integration into the electrical excitation device, which was based on a single tunneling heterojunction design. They further demonstrated arrays of WS_2_ and WSe_2_ SPEs by using nanopatterned silica substrates [241]. The SPEs showed visible-range PL, with WSe_2_ emitting in the 730–820 nm range and WS_2_ typically in the 610–680 nm range. The SPEs also showed PL stability, with spectral wandering remaining below 0.5 meV over a period of 1–2 min. Klein et al. [248] patterned SPEs in monolayer MoS_2_ with helium ion irradiation and achieved emission at 705 nm and $g^{\left(2\right)}\left(0\right)$ = 0.23 at cryogenic temperatures. Yu et al. [252] demonstrated MoSe_2_ SPEs operating at 785 nm with $g^{\left(2\right)}\left(0\right)=0.29$ at cryogenic temperatures. Zhao et al. [38] demonstrated MoTe_2_ SPEs spanning the telecom region from 1,080 nm to 1,550 nm with $g^{\left(2\right)}\left(0\right)=0.058$ under pulsed excitation and $g^{\left(2\right)}\left(0\right)=0.181$ under CW excitation. Significant efforts have been made to raise the operating temperature of TMDC-based SPEs beyond the cryogenic range. Parto et al. [246] used a combination of strain engineering via nanoscale stressors and defect engineering via electron-beam irradiation to pattern WSe_2_ SPEs with $g^{\left(2\right)}\left(0\right)=0.05$ at 5 K, and $g^{\left(2\right)}\left(0\right)=0.27$ at 150 K.

The weak van der Waals (vdW) interactions between TMDC layers allow for incommensurate stacking, which can result from relative rotation between the layers or, in heterobilayers, from lattice mismatch. The interplay between lattice mismatch and interlayer electronic coupling leads to the formation of interlayer excitons (IXs), where the exciton’s wavefunction spans both layers. For type-II band alignment, IXs possess out-of-plane electric dipole moments, enabling Stark tuning over a broad spectral range. Compared to intralayer excitons, IXs exhibit extended radiative lifetimes of order hundreds of nanoseconds and microsecond-scale valley lifetimes due to reduced electron–hole overlap. Combined with localized strain fields and in-gap defect states, such IXs lead to single-photon emission. Zhao et al. [253] demonstrated SPEs based on Γ – defect IXs in MoS_2_/WSe_2_ heterobilayers and heterotrilayers using nanopillars with a gold substrate. The nanopillars were used to create strain fields and defects, while the gold substrate quenched PL from the homogeneous region. The SPEs emitted at 855–1,078 nm with $g^{\left(2\right)}\left(0\right)=0.01$ under pulsed excitation at 10 K. When the lattice mismatch and/or interlayer twist between the TMDC bilayers is small, a moiré lattice is formed with lattice constants of hundreds even thousands of times larger than the sublattice. This moiré lattice generates a periodic variation in the potential energy landscape, known as the moiré potential, ranging from a few millielectronvolts to tens of millielectronvolts [254]. Such a moiré potential can trap excitons, creating an array of moiré excitons that each serve as SPEs. Seyler et al. [229] demonstrated Moiré potential-trapped valley IXs in MoSe_2_/WSe_2_ heterobilayers, confirmed by PL and the *g*-factor measurements of the emitters (Figure 8c). Baek et al. [255] realized MoSe_2_/WSe_2_ heterobilayer SPEs operating at 885 nm with $g^{\left(2\right)}\left(0\right)=0.28$ . As IXs possess vertical dipoles, they demonstrated that the photon energy could be tuned (∼40 meV) via DC stark effect. Chuang et al. [256] demonstrated an improvement in ***P*** from 60 % to 92 % for WSe_2_ SPEs by adding a graphene cap layer that quenched the background PL through fast interlayer charge transfer, preventing radiative recombination via long-lived defect-bound exciton states.

Substantial work has gone into cavity enhancement of TMDC SPEs. For instance, Luo et al. [230] demonstrated WSe_2_ SPEs coupled to plasmonic cavities, achieving a Purcell factor up to 551 and an enhanced QY of up to 65 % (average 44 %) (Figure 8d). Sortino et al. [257] coupled WSe_2_ SPEs to GaP dielectric nano-antennas, demonstrating a 10^2^–10^4^ enhancement of the PL intensity. Von Helversen et al. [231] demonstrated WSe_2_ SPEs with $g^{\left(2\right)}\left(0\right)=0.036$ under pulsed excitation at cryogenic temperatures (Figure 8e). Unfortunately, many TMDC SPEs exhibit low ***I*** due to their high dephasing rate. Drawer et al. [232] demonstrated WSe_2_ SPEs with $g^{\left(2\right)}\left(0\right)$ = 0.047 and 2 % visibility by coupling to a tunable optical cavity (Figure 8f), although it is not clear at this stage how much improvement on that visibility can be expected in optimized photonic platforms.

There is currently limited literature describing TMDCs integrated into functional quantum devices. For instance, Gao et al. [258] employed WSe_2_ SPEs with $g^{\left(2\right)}\left(0\right)$ = 0.034 to emulate the BB84 protocol in a QKD setup, achieving click rates of up to 66.95 kHz. Several issues hinder the broader application of TMDC SPEs: most TMDC SPEs require cryogenic temperatures to maintain high ***P***. Von Helversen et al. [231] performed temperature-dependent PL studies and time-resolved response studies on SPEs in monolayer WSe_2_ and attributed the PL decay to energy transfer between the emitter and the metal, mediated by Förster interaction. The low ***I*** (2 % visibility), caused by the short dephasing time (∼20 ps), as investigated by Drawer et al. [232], was attributed to rapid surface-induced charge noise. This issue constrains their suitability for applications like boson sampling. The ***B*** and QY of SPEs in TMDC heterostructures are limited due to the electronic band structure and spatial charge separation of IXs [253], [259]. Spectral tuning techniques and coupling to photonic structures can help overcome these intrinsic limitations in materials and devices, although much work remains to properly leverage TMDCs in integrated photonic devices for practical quantum applications.

## 8 Spectral tuning of SPEs

**Figure 9:**
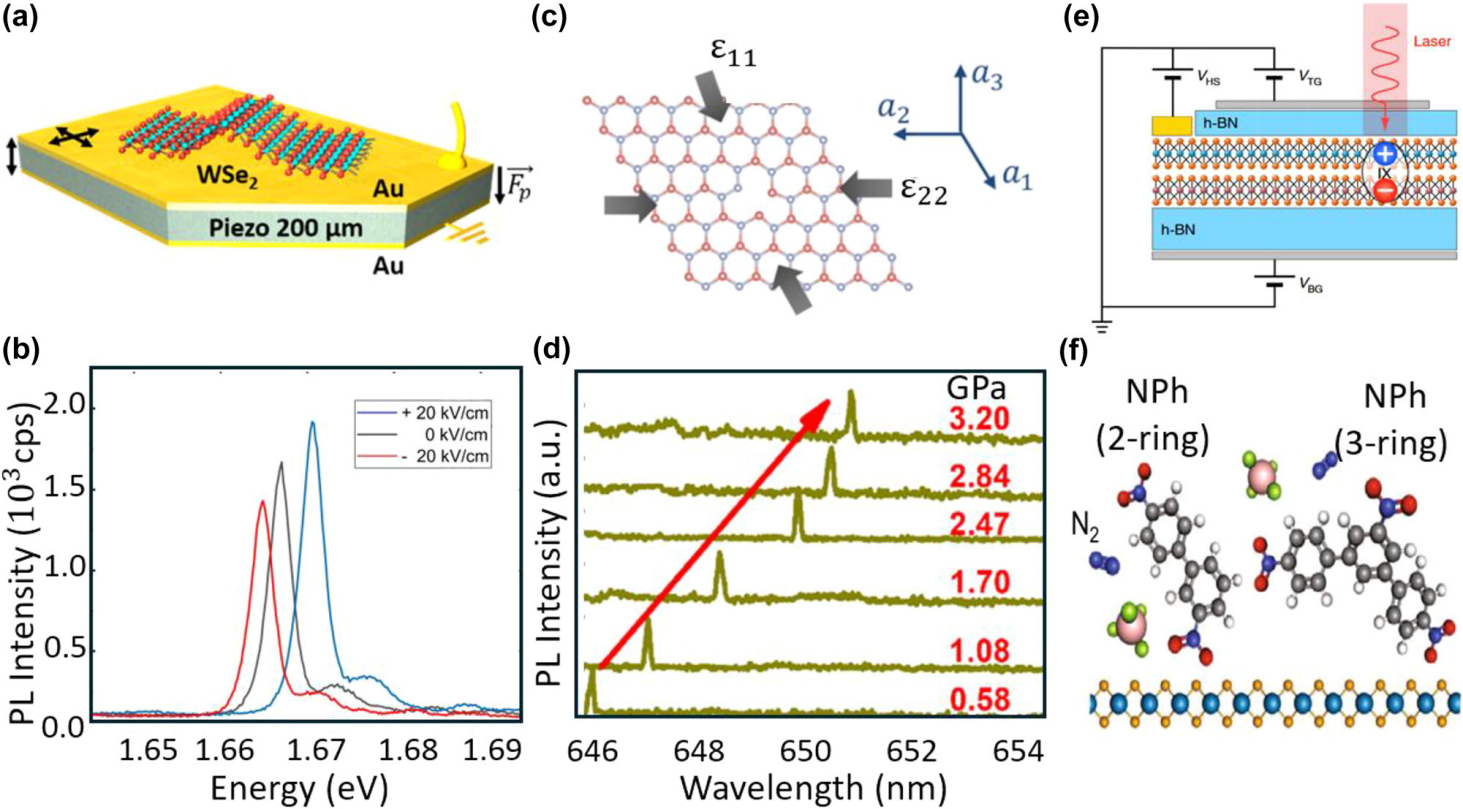
Spectral tuning of SPEs. (a) Schematic representation of a 2D hBN flake under strain. The strain components *ε*_11_ and *ε*_22_ are applied along two principal axes of the lattice, demonstrating the uniaxial or biaxial strain induced in the material. The crystallographic orientation is indicated by the axes *a*_1_, *a*_2_, and *a*_3_. (b) PL spectra of SPEs in the hBN flake under varying pressures, from 0.58 GPa to 3.20 GPa. The PL peak shows a redshift of ∼5 nm as the applied pressure increases, indicating strain-dependent spectral tuning. (c) Schematic representation of the experimental setup, featuring a WSe_2_ monolayer placed on a 200 µm piezoelectric substrate. Gold (Au) electrodes are used for electrical contact, and an external voltage is applied across the piezoelectric device to induce strain in the WSe_2_ layer, enabling spectral tuning. (d) PL spectra of the SPEs in WSe_2_ monolayer under different applied electric fields: +20 kV/cm (blue), 0 kV/cm (black), and −20 kV/cm (red). The shift in the PL peak energy with varying electric field demonstrates field-dependent control of emission properties. (e) Cross-sectional schematic of a heterostructure device comprising TMDC layers encapsulated by h-BN. The applied gate voltages (*V*_HS_, *V*_TG_, and *V*_BG_) generate an electric field across the TMDC heterostructure, enabling electrical tuning of interlayer excitons (IX) via the DC Stark effect. A laser excites the system, creating interlayer excitons, which are influenced by both the electric field and potential strain (indicated by *F*_*P*_) applied to the device. (f) Illustration of the chemomechanical modification process for SPEs in monolayer WSe_2_ using aryl diazonium chemistry. Treatment with 4-NBD results in the physisorption of a nitrophenyl (NPh) oligomer layer, consisting of 2-ring and 3-ring structures, onto the WSe_2_ surface. This functionalization suppresses strain-induced defect emissions, enabling the formation of spectrally isolated SPEs. Nitrogen gas (N_2_) is released as a by-product of the reaction. Adapted with permission from: (a), (b), ref. [261], Copyright 2018, American Chemical Society; (c), (d), ref. [262], Copyright 2019, American Chemical Society; (e), ref. [263], Springer Nature; (f), ref. [264], Springer Nature.

The reproducibility of LD-SPEs is limited by fabrication variability and material inhomogeneity. Spectral tuning can be achieved by strain engineering, the Stark effect, chemical functionalization, or combinations of these approaches. Spectral tuning via strain engineering was demonstrated in SPEs within 2D materials like hBN and TMDCs due to their high Young’s modulus (150–400 GPa). Grosso et al. [260] demonstrated that hBN SPEs can achieve spectral tunability of up to 6 meV by strain control using flexible polycarbonate (PC) beams. Xue et al. [261] measured the PL lines of hBN SPEs under varying hydrostatic pressure, demonstrating pressure coefficients of ∼15 meV/GPa at 5 K (Figure 9a and b). The SPEs exhibited a flexible bi-directional shift, showing both redshifts and blueshifts in response to pressure applied from different directions. Iff et al. [262] demonstrated a reversible tuning range of up to 18 meV in WSe_2_ SPEs using piezoelectric actuators (Figure 9c). They observed an energy shift of 5.4 μeV/V by sweeping the electric field applied to the piezoelectric actuator from −20 kV/cm to 20 kV/cm (Figure 9d).

Spectral tuning via the linear and quadratic Stark effect has been demonstrated in SPEs using hBN, TMDCs, and their heterostructures. The Stark effect is characterized by an energy shift Δℏ*ω*, which depends on the dipole moment $\vec{\mu }$ and polarizability $\vec{\alpha }$ of the system and is given by $\Delta \hbar \omega =-\Delta \vec{\mu }\cdot \vec{E}-\vec{E}\cdot \left(\frac{\Delta \vec{\alpha }}{2}\right)\cdot \vec{E}$ , where $\Delta \vec{\mu }$ is the difference in dipole moments between the excited and ground states and $\Delta \vec{\alpha }$ is the difference in polarizability between these states [265]. The first term represents the linear Stark shift, while the second term reflects the quadratic shift. Chakraborty et al. [266] studied the Stark effect of monolayer WSe_2_ SPEs in response to vertical electrical field, demonstrating a spectral tunability of up to 21 meV while varying the electric field from −100 MV/m to 400 MV/m. Noh et al. [265] investigated Stark effects of hBN/graphene SPEs in response to vertical field, demonstrating wavelength shifts as large as 5.4 nm per GV/m. Nikolay et al. [267] studied Stark effect of hBN SPEs in response to vertical field, demonstrating reversible wavelength shifts of (5.5 ± 0.3) nm around 670 nm by applying 20 V. Zhigulin et al. [268] studied Stark effects of blue hBN SPEs in response to lateral fields, demonstrating ${\left|\Delta \vec{\mu }\right|}_{\|}$ ∼ 0.1 D and $\left|\Delta \vec{\alpha }\right|$ ∼ 1,078 Å^3^. Ciarrocchi et al. [263] demonstrated electrical tuning of IXs in MoSe_2_/WSe_2_ heterostructures (Figure 9e), achieving an energy shift from 1.330 eV to 1.468 eV and a ${\left|\Delta \vec{\mu }\right|}_{\|}$ ∼ 24 D. Baek et al. [255] observed DC Stark effects in SPEs within MoSe_2_/WSe_2_ moiré heterobilayers, achieving a tuning range of ∼40 meV and a ${\left|\Delta \vec{\mu }\right|}_{\|}$ ∼ 21 D, with $g^{\left(2\right)}\left(0\right)=0.28$ at cryogenic temperature.

Spectral tuning via chemical functionalization was demonstrated in SPEs within SWCNTs and TMDCs. He et al. [74] demonstrated tunable wavelengths ranging from 1,280 nm to 1,550 nm in SWCNT SPEs at room temperature by varying the chiral index and aryl functionalization. Zhao et al. [33] demonstrated a wavelength shift in GQD SPEs, from approximately 650 nm to 760 nm after functionalization with chlorine atoms. Utama et al. [264] demonstrated a chemomechanical approach to modify the spectra of WSe_2_ SPEs (Figure 9f). They applied surface modification to strained monolayer WSe_2_ using 4-nitrobenzenediazonium (4-NBD) tetrafluoroborate. This process quenched most strain-induced defect emission, resulting in sharp SPEs with $g^{\left(2\right)}\left(0\right)=0.01$ after background correction and clean emission spectra and spectral tuning of approximately 20 meV.

## 9 Cavity coupling for SPE optimization

**Figure 10:**
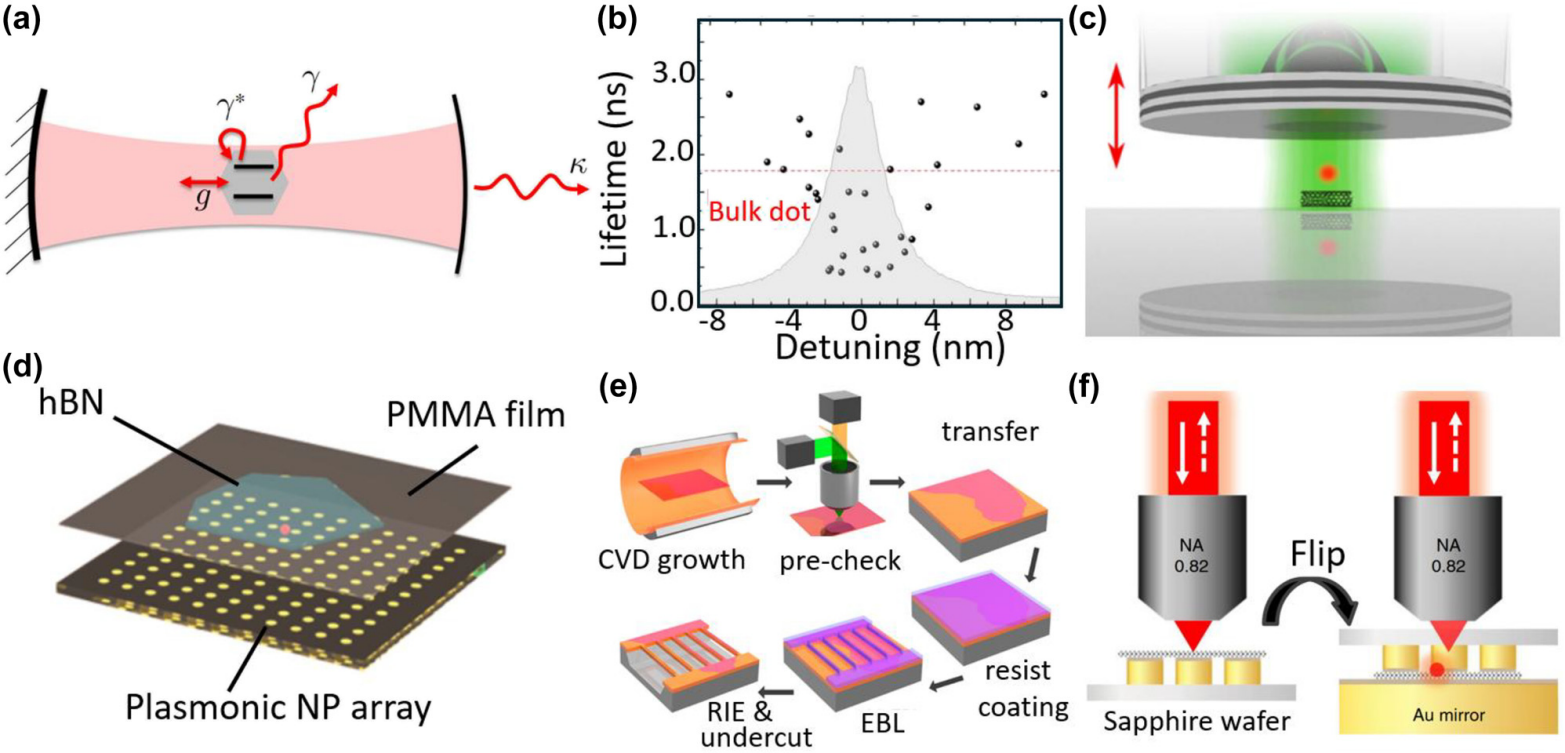
SPEs couple with cavities. (a) Schematic of a two-level emitter coupled to an optical cavity mode. The emitter experiences dissipation (*γ*) and interacts with the cavity mode via coupling strength *g*. Photons escape the cavity with decay rate *κ*, while *γ*∗ represents additional dissipation channels. (b) Lifetime measurements of 32 InAs/InP QDs from 20 different cavities, showing the impact of detuning on the QD lifetime. The shaded region represents the cavity effect, with the red dashed line indicating the lifetime of a bulk dot. (c) Schematic of a SWCNT coupled to a tunable microcavity. The cavity length is adjustable (indicated by the red arrows), allowing control over the optical coupling with the SPEs in the SWCNT. (d) Schematic of a hBN flake placed on a plasmonic nanoparticle array, covered by a poly (methyl methacrylate) (PMMA) film. (e) Fabrication process for integrating CVD-grown hBN with one-dimensional photonic crystal cavities. The steps include CVD growth, transfer, resist coating, electron beam lithography (EBL), and reactive ion etching (RIE) with undercutting. (f) Schematic of the experimental setup used to measure PL intensity and spontaneous emission rate from strain-induced SPEs in a WSe_2_ monolayer. Left: the emitters are positioned on gold pillars before the formation of the plasmonic nanocavity. Right: after the formation of the plasmonic nanocavity by flipping the material on a planar gold (Au) mirror, leading to enhanced emission properties. Adapted with permission from: (a), ref. [269], Copyright 2015 American Physical Society; (b), ref. [270], Copyright 2016 Optical Society of America; (c), ref. [271], Copyright 2017 American Chemical Society; (d), ref. [272], Copyright 2017, American Chemical Society; (e), ref. [221], Copyright 2020, American Chemical Society; (f), ref. [230], Springer Nature.

Coupling LD-SPEs to optical cavities enhances the ***B*** and ***I*** by introducing additional decay pathways and strong light–matter interactions due to electromagnetic confinement. As shown in Figure 10a, a two-level emitter coupled to an optical cavity mode can dissipate energy into the optical cavity via a Jaynes–Cummings type interaction [273], characterized by a coherent coupling rate (*g*). The cavity then releases the energy into the environment at a rate determined by the cavity linewidth (*κ*). The emission enhancement is quantified by the Purcell factor (*F*_*P*_), which is defined as $F_{P}=\frac{W_{\text{cav}}}{W_{\text{free}}}$ , where *W*_cav_ and *W*_free_ are the emission rates in the cavity and free space [104], respectively. The Purcell factor also depends on the quality factor (*Q*) and the mode volume (*V*) of the cavity, following the relation $F_{P}\propto \frac{Q}{V}$ . In the weak coupling regime, where *g* ≪ *κ*, the Purcell factor is given by $F_{P}=\frac{4g}{\kappa \gamma_{r}}$ . Cavity quantum electrodynamics (CQED) models for SPEs have been studied and discussed in numerous papers [269], [274].

A growing literature has focused on optimizing ***P*** and ***I*** in 0D QD SPEs and 1D SWCNT SPEs through integration with optical cavities. For instance, Kim et al. [270] coupled InAs/InP QD SPEs to nanophotonic cavities, demonstrating a visibility of 67 % after postselection. They measured the lifetimes of 32 QDs from 20 cavities, observing a lifetime as short as 400 ps for the fastest-emitting QD, corresponding to a *F*_*P*_ of 4.4 ± 0.5 (Figure 10b). Tomm et al. [96] coupled InGaAs EQD SPEs to a tunable Fabry–Pérot cavity, featuring a concave top mirror embedded in a silica substrate and a planar bottom mirror in the semiconductor heterostructure. They achieved $g^{\left(2\right)}\left(0\right)=0.02$ , visibility of 0.98, and an end-to-end efficiency of 0.57. Ding et al. [275] coupled InAs EQD SPEs to a similar microcavity, exhibiting high ***P*** (0.9795), ***I*** (0.9856), and efficiency of 0.712 at 4 K. Their open Fabry–Pérot cavity consisted of a concave top mirror made from 5.5 pairs of SiO_2_/Ta_2_O_5_ distributed Bragg reflector (DBR) layers, and a bottom mirror featuring a *λ*-thick quantum dot membrane on a 30-pair AlAs/GaAs DBR, with reflectivities of ∼98.6 % and ∼99.97 %, respectively. Farrow et al. [152] coupled CsPbBr_3_ PQD SPEs to an optical microcavity, realizing ***P*** of 0.94 and a narrow linewidth of approximately 1 nm. The custom open Fabry–Pérot microcavity features a small mode volume (<1 μm^3^), high *Q*-factors (>10^4^), and tunable wavelengths (450–950 nm), consisting of a planar mirror with spin-coated PQDs and a curved mirror, with positioning controlled by piezoelectric stages. Jun et al. [151] coupled CsPbBr_3_ PQD SPEs to a circular Bragg grating (CBG) cavity made by Si_3_N_4_ on silicon, realizing ultrafast (<100 ps) single-photon emission and 5.4-fold PL enhancement. Jeantet et al. [271], [276] coupled PFO-wrapped CoMoCat SWCNT SPEs to a fiber microcavity [277] (Figure 10c), achieving a *F*_*P*_ of up to 60 and a 20-fold PL enhancement at 20 K. The cavity features an asymmetric Fabry–Pérot structure with a 50 μm radius of curvature top mirror, mounted on an optical fiber using CO_2_ laser ablation, and a back mirror allowing 88 % photon transmission. Leveraging the flexibility of the fiber cavity, they achieved a ∼10 meV tunable emission energy by adjusting cavity detuning and explored unique exciton–phonon interactions, demonstrating an *I* of 0.25. Husel et al. [103] coupled SWCNT SPEs to a fiber cavity and operated the system in the regime of incoherent good cavity coupling [269], where photon coherence time is governed by the cavity linewidth. By choosing a cavity with a spectrally narrow linewidth, the SPEs exhibited HOM visibility of 0.65 at room temperature in the telecom band.

2D SPEs can conform closely with optical cavities, leading to a high *F*_*P*_. Tran et al. [272] demonstrated the deterministic coupling of hBN SPEs to plasmonic nanocavity arrays (Figure 10d), exhibiting 2.6-fold PL enhancement and $g^{\left(2\right)}\left(0\right)=0.29$ . The hBN flakes containing precharacterized SPEs were transferred onto the plasmonic nanoparticle arrays using a wet transfer method ensuring precise placement and strong coupling between the emitters and the plasmonic cavities. Fröch et al. [221] demonstrated the on-chip integration of CVD-grown hBN SPEs with one-dimensional photonic crystal nanobeam cavities fabricated from Si_3_N_4_ (Figure 10e), achieving a *Q* of 3,300 and a 6-fold PL enhancement. Nonahal et al. [70] coupled blue hBN SPEs to a 1D nanobeam photonic crystal cavity made from exfoliated hBN flakes, achieving a *Q* over 1,000, a *F*_*P*_ of 76, and a 4-fold PL enhancement at room temperature. Cai et al. [278] coupled monolayer WSe_2_ SPEs with the plasmon mode of a silver nanowire, achieving a lower-bound coupling efficiency of 26 % ± 11 %. Flatten et al. [279] coupled WSe_2_ SPEs to open microcavities, achieving a *F*_*P*_ of up to 8 and 5-fold PL enhancement. Their plano-concave microcavity consisted of two silica substrates, each coated with a DBR of 13 SiO_2_/TiO_2_ pairs, providing >99.95 % reflectivity at 740 nm. Luo et al. [230] demonstrated WSe_2_ SPE – plasmonic cavity systems with a *F*_*P*_ of up to 551. In this setup, the monolayer WSe_2_ was positioned onto gold pillars, with the hybrid structure flipped onto a smooth, thin layer of gold (Figure 10f).

## 10 Conclusions and perspectives

LD-SPEs offer high BPI values, various levels of scalability, room-temperature operation, telecom emission, electrically and optically driven emission, and fine spectral tunability. Key hosts for LD-SPEs include QDs, SWCNTs, hBN, and TMDCs, each with unique strengths and challenges based on their synthesis methods and physical properties. CQD SPEs, especially PQD SPEs, demonstrate near-unity QY, room-temperature operation, high ***P*** (0.98), and ***I*** (0.56) but are limited by low PL intensity due to strong Auger recombination. EQD SPEs show strong BPI values and scalability but are typically restricted to cryogenic temperatures. SWCNT SPEs offer high ***P*** (0.69) and ***I*** (0.65) at room temperature in the telecom band but are constrained by low QY and strong exciton–exciton annihilation (EEA). hBN SPEs have demonstrated reproducible wavelengths (i.e., 436 nm for blue emitters), high light collection efficiency (98 %), and ***I*** (0.56) at room temperature but suffer from low QY and nontelecom emission. TMDC SPEs have achieved high QY (65 %), ***P*** (0.95), and integration into photonic heterostructures, but they suffer from low ***I*** (2 %) due to high dephasing rates. Resonant excitation schemes [280] have potential to improve the ***I*** of hBN and TMDC SPEs. Spectral tuning using strain engineering, the Stark effect, and chemical functionalization is essential to fine tuning emission wavelengths, and LD-SPEs are especially well suited to manipulation by nanoscale photonic cavities.

SPE arrays that can generate many indistinguishable single photons simultaneously are an essential building block for photonic quantum computing. The performance of such systems is affected by factors including the BPI values (Table 1) and the number of photons contributing to multiphoton interference. For example, in Boson sampling, achieving interference with 50 photons and an error threshold of 10 % requires a photon indistinguishability greater than 94.7 % [119]. A critical challenge for scalable quantum technologies based on LD-SPEs is ensuring uniform emitter performance and deterministic emitter placement. However, the literature targeting uniform SPE performance is limited because it is challenging to manipulate the solid-state environment of LD-SPEs at the nanoscale. Blue hBN SPEs exhibit reproducible wavelengths at 436 nm [185], but the ***P*** and ***I*** vary among emitters. While there is substantial literature exploring deterministic SPE patterning by ion [189]/laser [281] irradiation, these SPEs still exhibit substantial variation from emitter to emitter because of stochastic changes to the defect environment induced by the patterning [191]. Beyond defects and strain fields, IXs confined in the periodic moiré potential across TMDC bilayers present a promising avenue for SPE arrays [259] enabled by the intrinsic spatial reproducibility of the moiré lattice; improved control of clean 2D interfaces will be essential to the realization of moiré quantum photonic platforms.

Many new materials are emerging as candidates for LD-SPEs. 2D metal monochalcogenides (MMCs), such as InSe [282], [283], [284], SnS, GaSe [285], [286], and GeSe [79], [287], exhibit a direct bandgap in both multilayer and bulk forms [288], [289], [290], enabling realizations of SPEs via strain or defect engineering that are compatible integrated photonic platforms [291]. Mixed-dimensional materials [292] combine the features of different dimensionalities, such as the scalability of 0D QDs with the valleytronics of 2D systems [293]. SPEs from such mixed-dimensional heterostructures offer potential for high ***P*** and scalability. Atomically thin perovskites [294] exhibit remarkable optoelectronic properties, including high photoluminescence efficiency and tunable emission wavelengths. SPEs can be realized in such materials via techniques used on 2D materials such as electron-beam and femtosecond-laser irradiation. Recently, heterostructures have been shown to generate chiral single-photon emission [295] and improve ***P***, offering new opportunities in quantum applications.

Despite substantial research efforts over the past decade and early demonstrations of quantum metrology [6], quantum computing [5], and quantum networking [296], most LD-SPEs have not yet reached the technological maturity of approaches based on QDs or heralded single photons from SPDC or SFWM processes. However, EQD SPEs have begun to reach the necessary thresholds in brightness, purity, and indistinguishability, which have led to a successful demonstration of 5-photon Boson sampling [116]. PQD SPEs and SWCNT SPEs also exhibit strong potential with their high ***I***, although their functionality in actual applications has not yet been explicitly demonstrated. Based on recent advanced with these materials, it is reasonable to expect EQD, PQD, and SWCNT SPEs to be integrated into boosted Bell state measurements [297] and repeaters [298] in the near future. The applications of 2D SPEs are more limited due to low ***I***, but the ***B*** and ***P*** achieved to date make them suitable for QKD applications [4], and further improvements in materials processing could unlock 2D SPEs for a broader range of quantum photonic technologies. The substantial progress in engineering LD spin defects over the past several years has unlocked new opportunities for spin-based quantum sensing, with a particular benefit offered by 2D spin defects in hBN that offer a dual purpose of encapsulating environmentally sensitive materials while hosting sensitive probes of local electric and magnetic fields. However, the literature focused on LD spin defects is still relatively new, and much work remains to determine the fundamental limits of these quantum sensors.

Beyond applications relying on emitted single photons, LD-SPEs may replace traditional solid-state SPEs, such as QD and color centers, in cavity-QED systems and enable novel designs that can be used as quantum memories [299], [300], [301], [302]. From an engineering perspective, LD-SPEs may be more compatible with integrated photonic cavities, but control over the SPE properties during photonic integration remains a technical challenge in many cases. The future development of LD-SPEs faces challenges in achieving desirable scalability, on-chip integration, and robustness. Ongoing advancements in theoretical research and fabrication techniques offer the potential to optimize LD-SPEs. As innovations in these areas keep enhancing efficiency and functionality, LD-SPEs are expected to unlock new possibilities across a broad range of applications, paving the way for transformative breakthroughs in quantum technologies and beyond.
